# Characterization of target gene regulation by the two Epstein-Barr virus oncogene LMP1 domains essential for B-cell transformation

**DOI:** 10.1128/mbio.02338-23

**Published:** 2023-11-27

**Authors:** Bidisha Mitra, Nina Rose Beri, Rui Guo, Eric M. Burton, Laura A. Murray-Nerger, Benjamin E. Gewurz

**Affiliations:** 1 Division of Infectious Diseases, Department of Medicine, Brigham and Women’s Hospital, Boston, Massachusetts, USA; 2 Center for Integrated Solutions for Infectious Disease, Broad Institute of Harvard and MIT, Cambridge, Massachusetts, USA; 3 Department of Microbiology, Harvard Medical School, Boston, Massachusetts, USA; The University of North Carolina at Chapel Hill, Chapel Hill, North Carolina, USA

**Keywords:** gammaherpesvirus, lymphoproliferative disease, tumor virus, B-cell oncogenesis, interferon regulatory factor, BATF, NF-κB, super-enhancer, dependency factor, apoptosis

## Abstract

The Epstein-Barr virus (EBV) oncogene latent membrane protein 1 (LMP1) mimics CD40 signaling and is expressed by multiple malignancies. Two LMP1 C-terminal cytoplasmic tail regions, termed transformation essential sites (TES) 1 and 2, are critical for EBV transformation of B lymphocytes into immortalized lymphoblastoid cell lines (LCL). However, TES1 versus TES2 B-cell target genes have remained incompletely characterized, and whether both are required for LCL survival has remained unknown. To define LCL LMP1 target genes, we profiled transcriptome-wide effects of acute LMP1 CRISPR knockout (KO) prior to cell death. To then characterize specific LCL TES1 and TES2 roles, we conditionally expressed wildtype, TES1 null, TES2 null, or double TES1/TES2 null LMP1 alleles upon endogenous LMP1 KO. Unexpectedly, TES1 but not TES2 signaling was critical for LCL survival. The LCL dependency factor cFLIP, which plays obligatory roles in blockade of LCL apoptosis, was highly downmodulated by loss of TES1 signaling. To further characterize TES1 vs TES2 roles, we conditionally expressed wildtype, TES1, and/or TES2 null LMP1 alleles in two Burkitt models. Systematic RNAseq analyses revealed gene clusters that responded more strongly to TES1 vs TES2, that respond strongly to both or that are oppositely regulated. Robust TES1 effects on cFLIP induction were again noted. TES1 and 2 effects on expression of additional LCL dependency factors, including BATF and IRF4, and on EBV super-enhancers were identified. Collectively, these studies suggest a model by which LMP1 TES1 and TES2 jointly remodel the B-cell transcriptome and highlight TES1 as a key therapeutic target.

**IMPORTANCE:**

Epstein-Barr virus (EBV) causes multiple human cancers, including B-cell lymphomas. In cell culture, EBV converts healthy human B-cells into immortalized ones that grow continuously, which model post-transplant lymphomas. Constitutive signaling from two cytoplasmic tail domains of the EBV oncogene latent membrane protein 1 (LMP1) is required for this transformation, yet there has not been systematic analysis of their host gene targets. We identified that only signaling from the membrane proximal domain is required for survival of these EBV-immortalized cells and that its loss triggers apoptosis. We identified key LMP1 target genes, whose abundance changed significantly with loss of LMP1 signals, or that were instead upregulated in response to switching on signaling by one or both LMP1 domains in an EBV-uninfected human B-cell model. These included major anti-apoptotic factors necessary for EBV-infected B-cell survival. Bioinformatics analyses identified clusters of B-cell genes that respond differently to signaling by either or both domains.

## INTRODUCTION

Epstein-Barr virus (EBV) is a gamma-herpesvirus that persistently infects most adults worldwide. EBV causes 200,000 cancers per year, including Burkitt lymphoma, Hodgkin lymphoma, post-transplant lymphoproliferative disease (PTLD), and HIV/AIDS-associated lymphomas. EBV also causes a range of epithelial cell tumors, including gastric and nasopharyngeal carcinomas, as well as T and NK cell lymphomas (1). The key EBV oncogene latent membrane protein 1 (LMP1) is expressed in most of these tumors, where it drives growth and survival pathway signaling.

To colonize the B-cell compartment and establish lifelong infection, EBV uses a series of viral latency genome programs, in which different combinations of latency genes are expressed. These include six Epstein-Barr nuclear antigens (EBNA) and the membrane oncoproteins LMP1, LMP2A, and LMP2B. LMP1 mimics aspects of signaling by the B-cell co-receptor CD40 (2–5), whereas LMP2A rewires surface B-cell immunoglobulin receptor signaling (6). All nine latency oncoproteins are expressed in the EBV B-cell transforming latency III program, which are expressed in immunoblastic lymphomas of immunosuppressed hosts. These include PTLD and primary central nervous system lymphoma. The latency II program is observed in EBV+ Hodgkin lymphoma, where the Reed-Sternberg tumor cells express EBNA1, LMP1, and LMP2A. Latency II is also frequently observed in T and NK cell lymphomas and in nasopharyngeal carcinoma. Host genome NF-κB activating mutations are frequently observed in EBV-negative Hodgkin lymphoma, but to a much lesser extent in EBV+ tumors, underscoring LMP1’s key role in activating growth and survival signaling (7).

LMP1 localizes to lipid rafts, where it signals constitutively in a ligand-independent fashion to activate NF-κB, MAP kinase, STAT3, PI3K, interferon, and P62 pathways. LMP1 is comprised of a short N-terminal cytoplasmic tail, six transmembrane (TM) domains, and a 200 residue C-terminal cytoplasmic tail (2–5). LMP1 TM domains drive homotypic aggregation, lipid raft association, and constitutive signaling (8, 9). The LMP1 C-terminal tail functionally mimics signaling from activated CD40 receptors, to the point that the CD40 tail can essentially be replaced by that of LMP1 in transgenic mice studies. However, while CD40/LMP1 knockin mice had relatively normal B-cell development and evidence of intact CD40 function, including germinal center formation and class switch recombination, T-cell independent B-cell activation was also observed (10). These experiments suggest that the LMP1 C-terminal tail mimics CD40 signaling but has also evolved additional functions.

Reverse genetic studies identified two LMP1 C-terminal cytoplasmic tail domains that are critical for EBV-mediated conversion of primary human B-cells into immortalized, continuously growing lymphoblastoid cell lines (LCL). Transformation effector site 1 (TES1), also called C-terminal activating region 1 (CTAR1), spans LMP1 residues 186–231. TES1/CTAR1 contains a PXQXT motif that engages tumor necrosis factor receptor associated factors (TRAFs). TES1 activates canonical NF-κB, non-canonical NF-κB, MAP kinase, and PI3K and STAT3 pathways (3, 4, 11–16). TES2, which spans residues 351–386 and is also referred to as CTAR2, activates canonical NF-κB, MAPK, IRF7, and P62 pathways (3–5, 16–20). TRAF6 is critical for LMP1 TES2-driven canonical NF-κB, MAPK, and p62 pathway activation (21–26). Canonical NF-κB signaling is critical for TES2/CTAR2-driven target gene regulation in a 293 cell conditional expression model (27). Signaling from a third LMP1 C-terminal tail region, CTAR3, activates JAK/STAT and SUMOylation pathways (28–30) potentially important *in vivo* but that are not essential for EBV-driven B-cell transformation (31). ChIP-seq analyses demonstrated a complex NF-κB binding landscape in LCLs, in which constitutive LMP1 signaling stimulates different combinations of the NF-κB transcription factors RelA, RelB, cRel, p50, and p52 to bind B-cell enhancers and promoters (32).

LMP1 is the only EBV oncogene that can independently transform rodent fibroblasts, driving anchorage-independent growth and loss of contact inhibition (33–35). Notably, CTAR1 signaling is sufficient for LMP1-mediated fibroblast transformation, whereas CTAR2 was dispensable (36). LMP1 expression drives aberrant B-cell growth in transgenic B-cell models, particularly in combination with LMP2 upon disruption of cell-mediated immunity (12, 37–40). While not critical for the first 8 days of EBV-driven B-cell outgrowth (41), LMP1 is critical for EBV-mediated conversion of primary human B-cells into immortalized LCLs (42, 43). A longstanding question has remained why TES1 and TES2 are each essential for EBV-mediated LCL establishment. Whether either or both are required for LCL survival is also unknown. Experiments using the EBV second-site mutagenesis method (44) demonstrated that TES1 is critical for initiation of EBV-infected lymphoblastoid cell outgrowth (45). By contrast, TES2 is critical for long-term LCL growth, although TES2 null EBV-infected lymphoblastoid cells could be propagated on epithelial feeders (45, 46). However, it remains incompletely understood the extent to which TES1 and TES2 play overlapping vs non-redundant roles.

While LMP1 B-cell target genes have been analyzed on small scales through qPCR and limited microarray analysis, unbiased genome-wide approaches have yet to be applied. Little is presently known about TES1 and TES2 shared vs non-redundant roles in transformed B-cells. To gain insights into LMP1 targets in the latency III LCL context, we therefore profiled transcriptome-wide changes in response to acute CRISPR LMP1 knockout (KO). These studies, performed at an early timepoint prior to apoptosis, identified that LMP1 strongly controls the LCL transcriptome, with expression levels of nearly 3,400 host genes significantly altered by LMP1 KO. To then characterize specific LCL TES1 and TES2 roles, we conditionally expressed wildtype, TES1 null, or TES2 null LMP1 rescue cDNAs at physiological levels upon endogenous LMP1 KO. This approach unexpectedly highlighted that signaling by TES1, but not TES2, is critical for LCL growth and survival. Loss of TES1 but not TES2 signaling rapidly triggered apoptosis, and strongly impaired expression of the LCL dependency factor cFLIP, which is required to block TNFα-driven apoptosis (25). Transcriptomic profiling further highlighted six clusters of LCL gene responses wildtype, TES1, or TES2 mutant LMP1, newly identifying independent, additive, or antagonistic roles in LCL target gene regulation. As multiple latency III genes often target the same host cell targets, we also constructed Burkitt B-cell models with conditional expression of wildtype, TES1, and/or TES2 null LMP1. These studies extended the LCL findings by further identifying shared vs distinct LMP1 roles in B-cell target gene regulation. Collectively, these studies highlight a complex landscape of TES1 and TES2 target gene regulation, in which each controls expression levels of large numbers of B-cell targets.

## RESULTS

### CRISPR analysis of LCL LMP1 target genes

**Fig 1:**
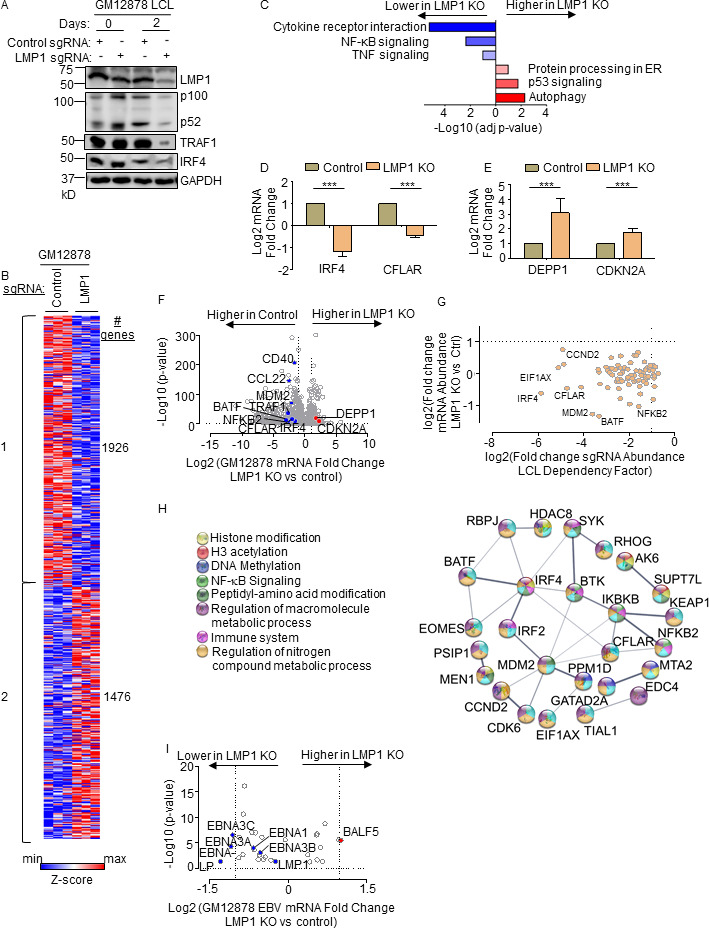
Characterization of LMP1 KO effects on GM12878 LCL target gene regulation. (**A**) Immunoblot analysis of whole cell lysates (WCL) from GM12878 LCLs transduced with lentiviruses that express control or LMP1 targeting single guide RNAs (sgRNAs). Transduced cells were puromycin selected for 0 vs 2 days, as indicated. Blots for LMP1, for LMP1 target genes TRAF1 and IRF4, and for LMP1-driven non-canonical NF-κB pathway p100/p52 processing are shown. Blots are representative of *n* = 3 experiments. (**B**) *K*-means heatmap analysis of GM12878 LCLs transduced as in panel **A** with lentivirus expressing control or LMP1 sgRNA and puromycin selected for 48 h. The heatmap depicts relative *Z*-scores in each row from *n* = 3 independent RNAseq replicates, divided into two clusters. The *Z*-score scale is shown at bottom, where blue and red colors indicate lower vs higher relative expression, respectively. Two-way ANOVA *P*-value cutoff of <0.05 and >2-fold gene expression cutoffs were used. (**C**) Enrichr analysis of KEGG pathways most highly changed in GM12878 expressing control vs LMP1 sgRNA (LMP1 KO), as in panel **A**. The *x*-axis depicts the −log10 adjusted *P*-value (adj *P*-value) scale. The top three most enriched KEGG pathways are shown. (**D**) Abundances of two representative Cluster 1 genes from *n* = 3 RNAseq analyses in cells with control vs LMP1 sgRNA. *P*-values were determined by one-sided Fisher’s exact test. *P < 0.05, **P < 0.01. (**E**) Abundances of two representative Cluster 2 genes from *n* = 3 RNAseq analyses in cells with control vs LMP1 sgRNA. *P*-values were determined by one-sided Fisher’s exact test. ***P < 0.001. (**F**) Volcano plot analysis of host transcriptome-wide GM12878 genes differentially expressed in cells with control vs LMP1 sgRNA expression, as in panel **B**, using data from *n* = 3 RNAseq data sets. (**G**) Scatter plot cross comparison of log2 transformed fold change mRNA abundances in GM12878 expressing LMP1 vs control sgRNA (*y*-axis) vs log2 transformed fold change abundances of sgRNAs at day 21 vs day 1 post-transduction of GM12878 LCLs in a genome-wide CRISPR screen (25) (*x*-axis). (**H**) String analysis of genes shown in panel **G**. Pathway identifiers for each gene and interaction are colored coded. (**I**) Volcano plot analysis of EBV mRNA values in GM12878 expressing LMP1 vs control sgRNAs, as in panel **B**. *P*-value < 0.05 and >2-fold change mRNA abundance cutoffs were used.

To characterize LMP1 target genes in the latency III context, we used CRISPR to knockout (KO) LMP1 in the well-characterized LCL GM12878, a Tier 1 Encode project cell line that we have used extensively for CRISPR analyses, and which we confirmed to have the latency III program (25, 47). GM12878 with stable Cas9 expression were transduced with lentivirus expressing a control single guide RNA (sgRNA) targeting a human genome intergenic region or LMP1. Immunoblot confirmed efficient LMP1 depletion by 48 h post-puromycin selection of transduced LCLs (Fig. 1A). CRISPR LMP1 editing rapidly downmodulated the LMP1/NF-κB target genes TRAF1 and IRF4 and decreased non-canonical pathway processing of the p100 NF-κB precursor into the active p52 transcription factor subunit, suggesting successful on-target effects of LMP1 knockout (KO) (Fig. 1A). At this early timepoint post-CRISPR editing, LCLs remained viable (Fig. S1A). However, LMP1 KO triggers LCL growth arrest and cell death shortly thereafter. We, therefore, used this early 2-day post-puromycin selection timepoint to perform systematic RNAseq analyses of control vs LMP1 KO LCLs. At a multiple hypothesis testing adjusted *P*-value <0.05 and fold change of >2 cutoff, acute LMP1 KO significantly altered the levels of around 3,400 host genes.

Genes differentially expressed in LMP1 KO vs control cells could broadly be characterized into two *k*-means clusters, in which LMP1 KO either downregulated 1,926 or upregulated 1,476 host genes (Fig. 1B; Table S7). Kyoto Encyclopedia of Genetic Elements (KEGG) pathway Enrichr analysis (48) identified that cytokine receptor signaling, NF-κB signaling, and tumor necrosis factor (TNF) signaling as enriched among genes rapidly downmodulated by LMP1 KO (Fig. 1C). As examples of cluster 1 NF-κB target genes, interferon regulatory factor 4 (IRF4) and CFLAR, which encodes c-FLIP, were strongly downmodulated by LMP1 KO (Fig. 1D). By contrast, KEGG highlighted that autophagy, p53 signaling, and protein-processing in the endoplasmic reticulum as enriched among cluster 2 genes (Fig. 1C). As examples from these enriched pathways, LMP1 KO highly induced expression of the autophagy suppressor DEPP1 and the p53 target and tumor suppressor cyclin-dependent kinase inhibitor 2A (CDKN2A) (Fig. 1D through F). Quantitative real-time PCR (qRT-PCR) validated RNAseq results for the CCL22, EBI3, and IRF4 mRNAs, each of which was significantly downmodulated by LMP1 KO (Fig. S1B through D).

We next integrated our RNAseq data set with published CRISPR analysis of host dependency factors essential for EBV+ LCL, but not Burkitt B-cell proliferation (25), to gain insights into key LMP1 roles in LCL growth and survival. This analysis identified that mRNA abundances of 37 of the 87 CRISPR-defined LCL selective dependency factors significantly changed upon LMP1 KO, suggesting multiple LMP1 roles in support of LCL survival (Fig. 1G; Fig. S1E). Of these, it is notable that multiple key suppressors of LCL intrinsic and extrinsic apoptotic pathways were rapidly lost upon LCL LMP1 KO. For instance, our published CRISPR analyses highlighted non-redundant roles for the transcription factors IRF4 and BATF in blockade of the intrinsic apoptosis pathway and for CFLAR-encoded cFLIP in extrinsic apoptosis pathway inhibition (25), each of whose mRNAs rapidly decreased upon LCL LMP1 KO. Likewise, LMP1 KO strongly downmodulated expression of MDM2, an LCL-selective dependency factor (25) that targets p53 for proteasomal degradation and that prevents LCL p53-dependent apoptosis (49). Furthermore, NF-κB blockade triggers LCL apoptosis (50), and the LCL dependency factor NFKB2, which encodes the NF-κB transcription factor subunit p52, was also highly downmodulated by LMP1 KO (Fig. 1F and G). STRING network analysis also underscored that each of these assembles into a network with 23 other LMP1-regulated LCL dependency factors (Fig. 1H).

Since LMP1 is highly expressed in Hodgkin lymphoma Reed-Sternberg tumor cells, we next analyzed effects of LMP1 KO on Hodgkin lymphoma KEGG pathway genes (Fig. S1F). Interestingly, LMP1 KO strongly downmodulated expression of the T-cell tropic chemokines CCL22 and CCL17, consistent with several prior reports linking LMP1 to their expression (51, 52). These findings raise the possibility that LMP1-driven chemokine expression may contribute to the striking enrichment of T-cells characteristic of the Hodgkin Reed-Sternberg microenvironment. However, volcano plot analysis also highlighted that LMP1 KO increased expression of CD274, which encodes the checkpoint inhibitor PD-L1, further implicating LMP1 in T-cell regulation.

Given widespread effects of LMP1 KO on LCL host gene expression, we next characterized effects of LMP1 KO on viral latency III genes. Mapping of RNAseq reads onto the GM12878 EBV transcriptome identified that LMP1 depletion significantly downmodulated mRNAs encoding EBNA3A, 3C, and EBNA-LP though interestingly not those encoding EBNA2 or EBNA3B (Fig. 1I). While it has been reported that LMP1 regulates its own mRNA expression (53, 54), we did not observe changes in LMP1 mRNA abundance upon LMP1 CRISPR KO. We note that CRISPR editing often results in insertions or deletions, causing functional protein knockout without necessarily changing mRNA levels of the edited gene. However, it is plausible a compensatory response to LMP1 knockout occurred on the mRNA level at this early timepoint, potentially balancing loss of NF-κB induced LMP1 expression. Taken together, our RNAseq analyses raise the possibility that secondary effects of LMP1 KO on Epstein-Barr nuclear antigens may also contribute to changes in the host transcriptome and cell death upon LMP1 KO.

### TES1 but not TES2 signaling is critical for LCL survival

**Fig 2:**
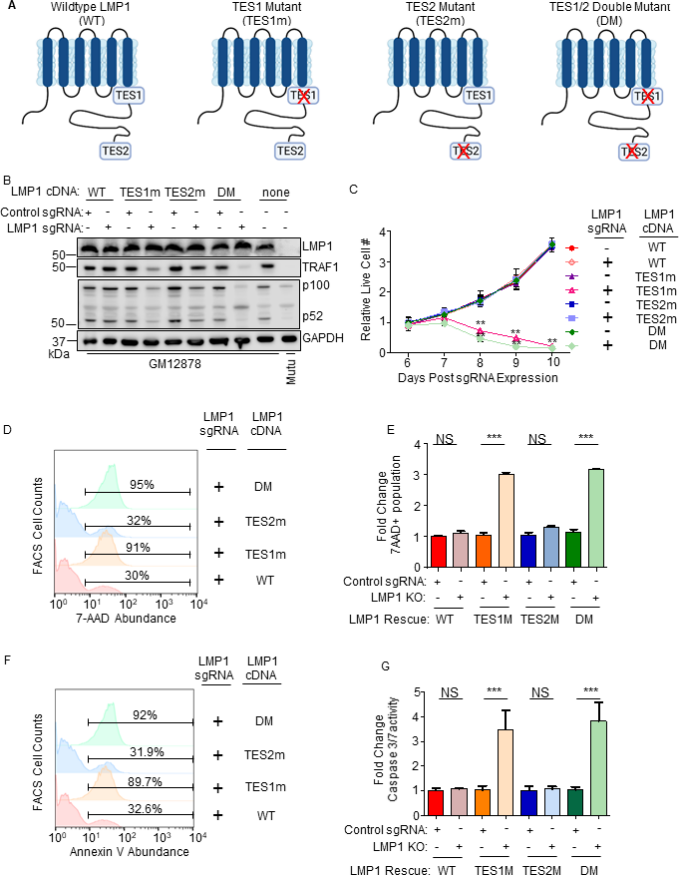
Loss of TES1 but not TES2 signaling triggers LCL apoptosis. (**A**) Schematic diagram of LMP1 WT with TES1 and TES2 domains highlighted. Wild-type (WT) or point mutants abrogated for signaling from TES1 (TES1m), TES2 (TES2m), or double TES1/TES2 mutant (DM) are shown. (**B**) Immunoblot analysis of WCL from GM12878 LCLs that expressed control or LMP1 sgRNAs and puromycin selected for 3 days were induced for expression with the indicated LMP1 rescue cDNA construct for 6 days. Blots are representative of *n* = 3 experiments. (**C**) Growth curve analysis of GM12878 LCLs at the indicated day post expression of control or LMP1 sgRNAs and the indicated LMP1 WT, TES1m, TES2m, or DM rescue cDNA. Shown are mean ± SD from *n* = 3 independent experiments. **P < 0.01. (**D**) FACS analysis of 7-AAD vital dye uptake in GM12878 on day 7 post-expression of LMP1 sgRNAs and the indicated LMP1 rescue cDNA. Shown are percentages of 7-AAD+ cells within the indicated gates. Representative of *n* = 3 experiments. (**E**) Mean ± SD of fold change 7-AAD values from *n* = 3 independent experiments of GM12878 with the indicated control or LMP1 sgRNA and rescue cDNA expression, as in panel **D**. Values in GM12878 with control sgRNA and no LMP1 rescue cDNA were set to 1. (**F**) FACS analysis of plasma membrane annexin V abundance in GM12878 on day 7 post-expression of control or LMP1 sgRNAs and the indicated LMP1 rescue cDNA. Shown are percentages of annexin V+ cells within the indicated gates. Representative of *n* = 3 experiments. (**G**) Mean ± SD of fold change caspase 3/7 activity levels, as determined by caspase 3/7 Glo assay, from *n* = 3 independent experiments of GM12878 with the indicated control or LMP1 sgRNA and rescue cDNA expression. Values in GM12878 with control sgRNA and no LMP1 rescue cDNA were set to 1.

While TES1 and TES2 signaling are each critical for B-cell transformation, it has remained unknown whether either or both are necessary for proliferation of fully transformed LCLs. Likewise, knowledge has remained incomplete about shared vs non-redundant TES1 and TES2 roles in LCL host gene regulation. To gain insights into these key questions, we engineered Cas9+GM12878 LCLs with conditional expression of wild-type (WT) LMP1, or with well-characterized point mutants that abrogate signaling from the TES1 TRAF-binding domain (TES1m, residues 204PQQAT208 → AQAAT), from TES2 (TES2m, 384YYD386 → ID) (46, 55, 56) (Fig. 2A). A silent mutation in the CRISPR protospacer adjacent motif (PAM) was used to abrogate CRISPR editing of these LMP1 rescue cDNA constructs. For cross-comparison, we also established conditional TES1/TES2 double-mutant (DM) cell lines with both mutations, to profile responses to other LMP1 regions, potentially including CTAR3 or unfolded protein responses induced by LMP1 induction (57, 58) (Fig. 2A). LCLs were then transduced with lentivirus expressing a control sgRNA targeting a human intergenic region or LMP1. Conditional LMP1 expression was then induced by addition of 400 ng/mL doxycycline, such that the rescue cDNA was induced as endogenous EBV-encoded LMP1 was depleted. We confirmed similar levels of LMP1 expression across this series and achieved similar LMP1 levels as in unedited GM12878 LCLs (Fig. 2B). Importantly, we validated that WT LMP1 rescued physiological levels of LMP1 target TRAF1 expression and p100/p52 processing (Fig. 2B). TES1 is responsible for the majority of LMP1-mediated TRAF1 induction and p100/p52 processing (55, 59), and as expected, conditional TES1m and DM expression failed to rescue physiological levels of TRAF1 or p100/p52 processing in GM12878 with endogenous LMP1 KO. By contrast, TES2m induced levels of TRAF1 and p100/p52 processing in LMP1 KO levels approaching those in unedited GM12878 (Fig. 2B), validating our LCL LMP1 KO/rescue system.

We next tested the effects of LMP1 WT, TES1m, TES2m, or DM rescue cDNA expression on GM12878 proliferation. Whereas signaling from both TES1 and TES2 is required for EBV-driven primary human B-cell growth transformation, we unexpectedly found that LCLs require signaling only from TES1 for growth and survival: LMP1 KO LCLs with WT vs TES2m rescue cDNA proliferated indistinguishably. By contrast, LCLs with TES1m or DM rescue cDNA expression failed to proliferate (Fig. 2C). To characterize this unexpected result further, we next measured effects of endogenous LMP1 KO and rescue LMP1 cDNA expression on LCL survival. Consistent with our growth curve analysis, LMP1 KO LCLs with TES1m or DM rescue cDNA exhibited widespread cell death, as judged by uptake of the vital dye 7-aminoactinomycin D (7-AAD) by FACS analysis (Fig. 2D and E). Consistent with apoptosis as the cell death pathway triggered by loss of TES1 signaling in LCLs, levels of annexin V and executioner caspase 3/7 activity were significantly higher in LMP1 KO GM12878 with TES1m or DM than with WT or TES2m rescue cDNA expression (Fig. 2F and G; Fig. S2). Taken together, these data newly suggest that TES1 signaling is necessary for LCL growth and survival in a manner that is not redundant with TES2 and that cannot be rescued by TES2 signaling alone.

### Identification of TES1 vs TES2 roles in LCL gene regulation

**Fig 3:**
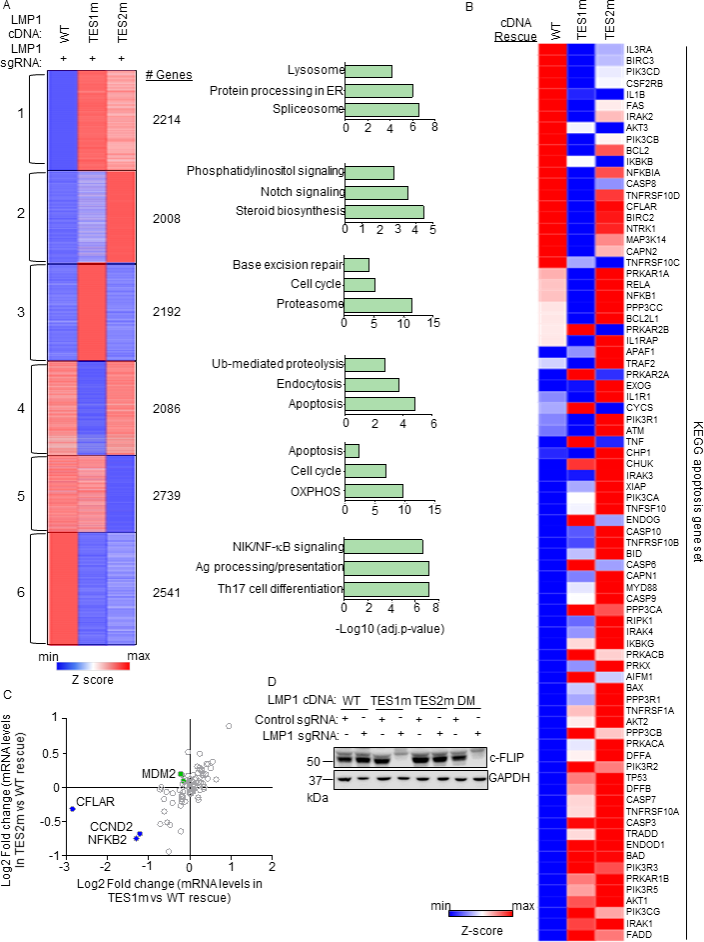
Characterization of host genome-wide TES1 vs TES2 LCL target genes. (**A**) RNAseq K-means heatmap analysis of GM12878 LCLs transduced with lentivirus expressing LMP1 sgRNA and induced for WT, TES1m, or TES2m rescue cDNA expression for 6 days. The heatmap depicts relative *Z*-scores in each row from *n* = 3 independent RNAseq data sets, divided into six clusters. The *Z*-score scale is shown at bottom, where blue and red colors indicate lower vs higher relative expression, respectively. Two-way ANOVA *P*-value cutoff of <0.05 and >2-fold gene expression cutoffs were used. The top three most highly enriched KEGG pathways amongst genes within each cluster are shown at right. (**B**) Heatmap analysis of KEGG apoptosis pathway gene relative row *Z*-scores from RNAseq analysis as in panel **A**. The *Z*-score scale is shown at bottom, where blue and red colors indicate lower vs higher relative expression, respectively. Two-way ANOVA *P*-value cutoff of <0.05 and >2-fold gene expression cutoffs were used. (**C**) Scatter plot analysis cross comparing log2 transformed fold change of LCL dependency factor mRNA abundances in GM12878 expressing LMP1 sgRNA together with TES2 mutant vs wild-type cDNA rescue (*y*-axis) and TES1 mutant vs wild-type cDNA rescue (*x*-axis) from triplicate RNAseq data sets, as in panel **A**. This analysis highlighted that CFLAR and to a lesser extent NFKB2 and CCND2 mRNAs were more highly downmodulated by TES1m than TES2m rescue, relative to levels in cells with WT LMP1 rescue. Shown are genes differentially regulated by >2-fold with either TES1m or TES2m rescue, relative to levels with WT LMP1 rescue. (**D**) Immunoblot analysis of c-FLIP and load control GAPDH expression in WCL from GM12878 LCLs with the indicated control or LMP1 sgRNA and LMP1 rescue cDNA expression. Representative of *n* = 3 experiments.

To gain insights into overlapping vs non-redundant TES1 and TES2 LCL roles, we performed biological triplicate RNAseq analyses to cross-compare GM12878 transcriptomes at day 6 post endogenous LMP1 KO and with doxycycline-induced WT, TES1m, or TES2m rescue cDNA expression. We selected this early timepoint as it is just prior to the divergence of the growth curves (Fig. 2C; Tables S8 and 9) and the onset of apoptosis. *K*-means analysis identified six clusters in which host gene expression differed between LMP1 KO LCLs with WT, TES1m, or TES2m cDNA rescue. KEGG analysis highlighted pathways most highly enriched in each cluster (Fig. 3A). Notably, apoptosis pathway genes were the most highly enriched in cluster 4, which were expressed at lower levels in cells with TES1m than with WT or TES2m rescue cDNA expression, suggesting that TES1 signaling may induce their expression. Apoptosis genes were also enriched among cluster 5 genes, where levels were lower in cells with TES2m expression, suggesting that TES2 signaling may induce their expression (Fig. 3A).

Given this apoptosis signal, we next analyzed the KEGG apoptosis gene set responses to WT, TES1m, or TES2m cDNA rescue (Fig. 3B). Interestingly, a cluster of genes were more highly expressed in cells with WT and TES2m than with TES1m expression, including the anti-apoptotic genes CFLAR, BCL2, and BIRC3, which encodes the cIAP2 ubiquitin ligase, which counteracts TNF-driven cell death. While BCL2 and BIRC3 were not defined as LCL dependency factors by genome-wide CRISPR analysis previously, CLFAR was (25). Intriguingly, while a small number of the 87 LCL dependency factors (25) were more highly downmodulated in LMP1 KO LCLs with TES1 rescue than with TES2 rescue, CFLAR was the LCL dependency factor most highly depleted by TES1m rescue relative to levels in TES2m rescue cells (nearly eightfold lower in TES1m rescue cells) (Fig. 3C; Fig. S3A). By contrast, CFLAR was only mildly depleted (<0.5-fold) in cells with TES2m rescue (Fig. 3C). We validated that CFLAR-encoded c-FLIP was highly downmodulated on the protein level in LMP1 KO LCLs with TES1m or DM LMP1 rescue but not in LCLs with WT or TES2m rescue at an early timepoint prior to cell death (Fig. 3D). The LCL dependency factors NFKB2 and CCND2 were also more highly downmodulated in LMP1 KO cells with TES1 than TES2 cDNA rescue, but not to the same extent as CFLAR (Fig. 3C), suggesting that their loss may not be responsible for apoptosis in the absence of TES1 signaling. Collectively, these analyses underscore distinct TES1 vs TES2 signaling roles in control of LCL apoptosis pathway gene expression.

To gain further insights into potential TES1 vs TES2 roles in regulation of genes with relevance to Hodgkin Reed-Sternberg cells, we analyzed KEGG Hodgkin lymphoma pathway gene expression in LMP1 KO GM12878 rescued with WT, TES1m, or TES2m LMP1. This analysis highlighted that many KEGG Hodgkin lymphoma pathway genes are jointly induced by TES1 and TES2 signaling in LCLs, including CCL22, BCL3, cRel, IRF4, STAT3, STAT6, and CD70, each of which has prominent roles in Hodgkin lymphoma pathogenesis (Fig. S3B). We validated RNAseq results for CCL22, EBI3 and IRF4 by qRT-PCR, again finding that TES1 and TES2 were jointly responsible for their expression (Fig. S3C through E). Interestingly, the Hodgkin lymphoma therapeutic target CD27 was expressed at lower level with TES1m rescue but more highly expressed in cells with TES2m rescue, suggesting that TES1 and TES2 signaling may jointly balance its expression.

**Fig 4:**
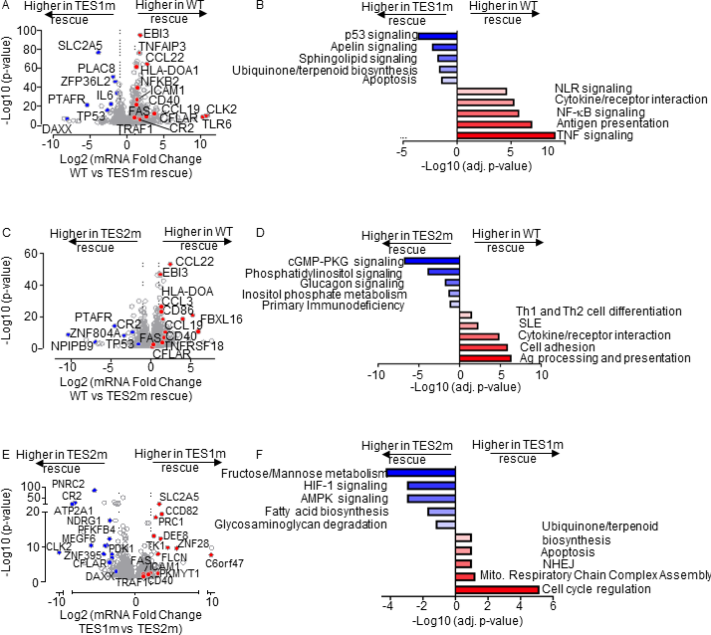
Characterization of LCL pathways targeted by TES1 vs TES2 signaling. (**A**) Volcano plot analysis of host transcriptome-wide GM12878 genes differentially expressed in LMP1 KO GM12878 with WT vs TES1 mutant cDNA rescue. Higher *x*-axis fold changes indicate higher expression with WT LMP1 rescue, whereas lower *x*-axis fold changes indicate higher expression with TES1m rescue. Data are from *n* = 3 RNAseq data sets, as in Fig. 3. (**B**) Enrichr analysis of KEGG pathways most highly enriched in RNAseq data as in panel **A** among genes more highly expressed in LMP1 KO GM12878 with WT than TES1m rescue (red) vs among genes more highly expressed with TES1m than WT rescue (blue). (**C**) Volcano plot analysis of host transcriptome-wide GM12878 genes differentially expressed in LMP1 KO GM12878 with WT vs TES2 mutant cDNA rescue. Higher *x*-axis fold changes indicate higher expression with WT LMP1 rescue, whereas lower *x*-axis fold changes indicate higher expression with TES2m rescue. Data are from *n* = 3 RNAseq data sets, as in Fig. 3. (**D**) Enrichr analysis of KEGG pathways most highly enriched in RNAseq data as in panel **C** among genes more highly expressed in LMP1 KO GM12878 with WT than TES2m rescue (red) vs among genes more highly expressed with TES2m than WT rescue (blue). (**E**) Volcano plot analysis of host transcriptome-wide GM12878 genes differentially expressed in LMP1 KO GM12878 with TES1 vs TES2 mutant cDNA rescue. Higher *x*-axis fold changes indicate higher expression with TES1m rescue, whereas lower *x*-axis fold changes indicate higher expression with TES2m rescue. Data are from *n* = 3 RNAseq data sets, as in Fig. 3. (**F**) Enrichr analysis of KEGG pathways most highly enriched in RNAseq data as in panel **E** amongst genes more highly expressed in LMP1 KO GM12878 with TES1m than TES2m rescue (red) vs among genes more highly expressed with TES2m than TES1m rescue (blue).

On the transcriptome-wide level, multiple well-characterized LMP1 target genes were more highly expressed in LCLs rescued with WT LMP1 than with TES1m cDNA, establishing these as key TES1 LCL target genes. These included CD40, TRAF1, EBI3, and ICAM1 (Fig. 4A), which were previously established as TES1 target genes in studies of cell lines overexpressing LMP1, including BL-41 Burkitt and BJAB diffuse large B-cell lymphoma models (55). Notably, this approach also newly suggests a large number of B-cell targets whose upregulation or downregulation is dependent on TES1 signaling. These include CFLAR and TLR6 (which encodes Toll-like receptor 6), which were significantly more highly expressed in LMP1 KO LCLs with WT LMP1 cDNA rescue. By contrast, the mRNA encoding the histone loader DAXX, which can serve as an epigenetic suppressor of EBV gene expression (60, 61) and DNA damage pathway TP53 (which encodes p53), were expressed at considerably higher levels in LMP1 KO LCLs with TES1m than WT LMP1 rescue cDNA (Fig. 4A). This result suggests that TES1 signaling may repress their expression. Enrichr analysis of genes more highly expressed with WT LMP1 rescue highlighted TNF and NF-κB signaling as enriched KEGG pathways, whereas p53 signaling and apoptosis were among the pathways most highly enriched in genes more highly expressed with TES1m rescue (Fig. 4B).

Volcano plot and KEGG pathway analysis highlighted LCL genes differentially expressed in LMP1 KO LCLs with WT vs TES2m rescue (Fig. 4C and D). KEGG pathways enriched among genes more highly expressed with WT LMP1 rescue again included antigen presentation and cytokine/receptor interaction, but also included systemic lupus erythematosus (SLE). Pathways enriched among genes more highly expressed with TES2m rescue instead included cyclic GMP protein kinase G (cGMP-PKG) signaling and phosphatidylinositol signaling (Fig. 4D). Notably, TP53 (which encodes p53) was also more highly expressed in LMP1 KO LCLs with TES2m than WT LMP1 rescue, suggesting that both TES1 and 2 signaling regulate its expression. By comparison, CFLAR expression was similar in LMP1 KO LCLs with WT and TES2m rescue, further establishing it as a TES1 target in LCLs (Fig. 4C).

Direct cross-comparison of genes in LCLs with TES1m vs TES2m rescue further identified roles of TES1 vs TES2 signaling on LCL target gene expression. The oncogenic kinase CLK2, which has roles in splicing regulation, was the host gene most highly expressed in LMP1 KO with TES2m versus in cells with TES1m rescue, newly indicating that it is strongly induced by TES1 or inhibited by TES2 signaling (Fig. 4E). Enrichr analysis indicated that multiple KEGG metabolism pathways were the most highly enriched in cells with TES2m rescue, including fructose/mannose metabolism, HIF1 signaling, and AMPK signaling (Fig. 4E and F). In support, the glycolytic enzyme PFKFB4 and the kinase PDK1, which regulates flux of glycolytic products to mitochondrial metabolism pathways at the level of pyruvate, were more highly expressed with TES2m rescue, suggesting that they are either driven by TES1 or repressed by TES2 signaling (Fig. 4F). Cell cycle regulation was the KEGG pathway most enriched among genes more highly expressed with TES1m rescue. The cyclin-dependent kinase (CDK) substrate and cytokinesis regulator PRC1, as well as the CDK1 kinase and mitosis regulator PKMYT1, were among the genes most highly differentially expressed in TES1m rescue (Fig. 4E and F), suggesting that TES2 drives or that TES1 instead inhibits their expression.

### B-cell genes induced by conditional expression LMP1 in EBV-negative Burkitt cells

As a complementary approach to our loss-of-function CRISPR KO LCL analyses, we next profiled B-cell responses to conditional LMP1 expression. A goal of this approach was to identify LMP1-specific effects on host gene expression since LMP1 KO significantly altered expression of several EBV latency III genes. Furthermore, EBNA and LMP latency III oncoproteins often jointly target host genes. Therefore, to study LMP1-specific effects in isolation of other latency III genes, we engineered EBV-negative Akata and BL-41 Burkitt B-cell lines with doxycycline-inducible WT, TES1m, TES2m, or DM LMP1 alleles. The Akata cell line was originally established from a human EBV+Burkitt tumor (62), but an EBV-negative subclone that spontaneously lost the viral genome was isolated shortly thereafter (63), which we used for these studies. Similarly, the BL-41 cell line was established from an EBV-negative human Burkitt lymphoma tumor (64). BL-41 were used for early microarray analysis of latency III or LMP1 effects on a subset of human genes (50). We validated that WT and point mutant LMP1 were expressed to similar extents across the panel. As expected, TES1m and DM exhibited impaired non-canonical NF-κB pathway activation, as judged by p100:p52 processing (Fig S4A and B). LMP1 signaling was also validated by FACS analysis of ICAM-1 and Fas upregulation. Consistent with a published study (55), TES1 signaling more strongly induced ICAM-1 and Fas in both Burkitt cell lines, even though BL-41 had somewhat higher basal NF-κB activity than Akata, as judged by Fas and ICAM-1 levels in uninduced cells (Fig. S4C through J).

**Fig 5:**
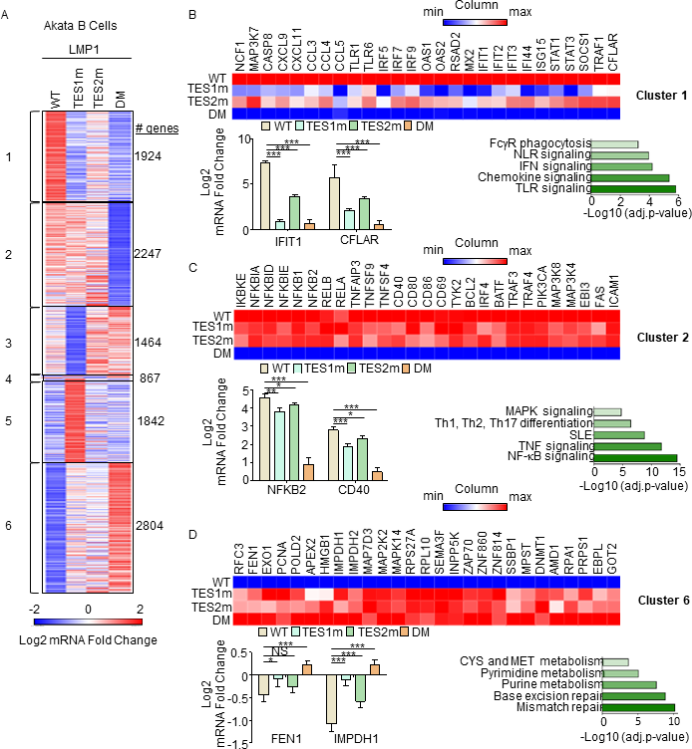
Characterization of host genome-wide Akata B-cell LMP1 target genes. (**A**) *K*-means heatmap analysis of RNAseq data sets from *n* = 3 replicates generated in EBV− Akata Burkitt cells with conditional LMP1 WT, TES1m, TES2m, or DM expression induced by 250 ng/mL doxycycline for 24 h. The heatmap visualizes host gene Log2 fold change across the four conditions, divided into six clusters. A two-way ANOVA *P* value cutoff of <0.01 and >2-fold gene expression were used. # of genes in each cluster is indicated at right. (**B**) Heatmaps of representative Cluster 1 differentially regulated genes (top), with column maximum (max) colored red and minimum (min) colored blue, as shown by the scalebar. Also shown are expression values of two representative Cluster 1 genes (lower left) and Enrichr analysis of KEGG pathways significantly enriched in Cluster 1 gene sets (lower right). *P*-values were determined by one-sided Fisher’s exact test. ***P < 0.001. (**C**) Heatmaps of representative Cluster 2 differentially regulated genes (top), as in panel **B**. Also shown are expression values of two representative Cluster 2 genes (lower left) and Enrichr analysis of KEGG pathways significantly enriched in Cluster 2 gene sets (lower right). *P*-values were determined by one-sided Fisher’s exact test. *P < 0.05, **P < 0.01, ***P < 0.001. (**D**) Heatmaps of representative Cluster 6 differentially regulated genes (top), as in panel **B**. Also shown are expression values of two representative Cluster 6 genes (lower left) and Enrichr analysis of KEGG pathways significantly enriched in Cluster 6 gene sets (lower right). *P*-values were determined by one-sided Fisher’s exact test. *P < 0.05, ***P < 0.001.

We then profiled effects of conditional WT, TES1m, TES2m, or DM expression for 24 h on the Akata transcriptome using biological triplicate RNAseq data sets. *K*-means heatmap analysis with *n* = 6 clusters revealed strikingly distinct patterns of host gene responses to WT, TES1m, TES2m, and DM LMP1 signaling (Fig. 5A; Tables S1 to S3). Cluster 1 genes were highly upregulated by WT LMP1, to a lesser extent by TES2m (in which only TES1 signals), and more modestly by TES1m (in which only TES2 signals). This result suggests that TES1 signaling contributes more strongly than TES2 to their expression. Notably, CFLAR was a Cluster 1 gene target, consistent with our finding that TES1 drives CFLAR expression in LCLs, as was the interferon stimulated gene IFIT1 (Fig. 5B). KEGG pathways enriched among Cluster 1 genes included TLR signaling, chemokine signaling, IFN signaling, and NLR signaling (Fig. 5B). Cluster 1 also contained well-described LMP1 target genes, including TRAF1, which we validated by immunoblot (Fig. S4A), consistent with a prior study (55). Notably, MAP3K7, which encodes the kinase TAK1, is also a Cluster 1 gene. Since TAK1 is critical for TES2/canonical NF-κB and MAP kinase signaling (26), this result suggests an important mechanism of cross-talk between TES1 and TES2. Likewise, the Cluster 1 gene product IRF7 binds to and is activated by TES2 (65–68), again suggesting cross-talk between LMP1 pathways. STAT1 and STAT3 are also Cluster 1 genes, raising the question of whether these STATs may drive interferon stimulated gene induction downstream of LMP1.

Cluster 2 genes were induced by WT, TES1m or TES2m LMP1 to a similar extent (Fig. 5C), suggesting that they redundantly respond to TES1 or TES2 signaling. KEGG pathway analysis highlighted enrichment of NF-κB signaling in this cluster, which included mRNAs encoding four NF-κB transcription factor subunits, as well as the NF-κB-induced inhibitors IκBα, IκBζ, and IκBε. mRNA fold changes for *NFBK2*, which encodes the non-canonical pathway NF-κB p52 transcription factor, are shown in Fig. 5C and are consistent with our LCL rescue analysis, which identified similarly important roles for both TES1 and TES2 in support of NFKB2 expression (Fig. 3C). Consistent with a prior study (55), Fas and ICAM-1 are Cluster 2 genes similarly induced on the mRNA level by TES1m and TES2m. However, plasma membrane ICAM-1 levels were lower in cells expressing TES1m (Fig. S4C through F). This result raises the possibility that TES1 signaling may play a role in ICAM-1 post-transcriptional regulation and/or trafficking.

Cluster 3 genes were expressed at lower levels in cells expressing TES1m, even as compared with cells expressing LMP1 DM, suggesting that unopposed TES2 signaling results in their downregulation (Fig. 5A; Fig. S5A). Cluster 3 genes were enriched for multiple KEGG metabolism pathways, including oxidative phosphorylation (Fig. S5A). Cluster 4 contained a smaller subset of host genes, downregulated by TES1 signaling, including in the WT LMP1 context. This gene set was enriched for SNARE interactions in vesicular transport and sphingolipid metabolism (Fig. S5B). Cluster 5 mRNAs were instead upregulated by unopposed TES2 signaling (Fig. 5A; Fig. S5C) and enriched for the KEGG ubiquitin-mediated proteolysis pathway. Finally, Cluster 6 genes were repressed by TES1 and TES2 signaling in an additive manner (Fig. 5D). This gene set was enriched for mismatch and base excision repair, nucleotide metabolism, cystine, and methionine metabolism. TES1 and TES2 signaling may additively recruit the same repressors or may instead recruit co-repressors to these sites. We validated effects on CCL22, EBI3, and IRF4 expression by qRT-PCR (Fig. S5D through F).

We next used RNAseq to profile BL-41 Burkitt cells. RNAseq was performed at 24 h post-expression of WT, TES1m, TES2m, or DM LMP1. *K*-means analysis with *n* = 6 clusters again revealed categories of genes that respond differently to LMP1 alleles (Fig. S6A; Tables S4 to S6). As observed in Akata, Cluster 1 genes were most highly induced by WT, and to a lesser extent by TES1m or TES2m, suggesting that TES1/2 additively or synergistically induce their expression. Cluster 1 genes contained multiple pro-inflammatory factors, including chemokines and the interferon pathway transcription factors STAT1, IRF4, IRF5, and IRF9 (Fig. S6A and B). As observed in Akata, Cluster 2 genes were induced more strongly by TES2m than by TES1m, and to a somewhat higher level by WT LMP1, suggesting that these are predominantly TES1 target genes (Fig. S6C). Consistent with our LCL and Akata cell analyses, CFLAR was a Cluster 2 gene more highly induced by LMP1 alleles with TES1 signaling, further underscoring it as a key TES1 target gene (Fig. S6C). By contrast, BL-41 Cluster 4 genes were instead suppressed by unopposed TES1 signaling even relative to levels observed in cells with LMP1 DM expression, suggesting that TES2 may block TES1 repressive effects on these host targets (Fig. S6D). Cluster 6 genes were enriched for the antigen presentation pathway and were most highly induced by TES2m, suggesting positive TES1 and potentially also negative TES2 roles in their induction (Fig. S6E). While concordant to a large degree, we speculate that observed differences between LMP1 effects on Akata vs BL-41 host gene expression may likely reflect the somewhat higher basal NF-κB levels observed in BL-41, and perhaps also differences in driver mutation pathways frequently found in EBV+ vs EBV− Burkitt lymphomas (69, 70). Nonetheless, both models highlight distinct clusters of host B-cell target genes that differ in responses to TES1, TES2, or combined TES1/2 signaling.

### LMP1 WT, TES1, TES2 and DM target genes

**Fig 6:**
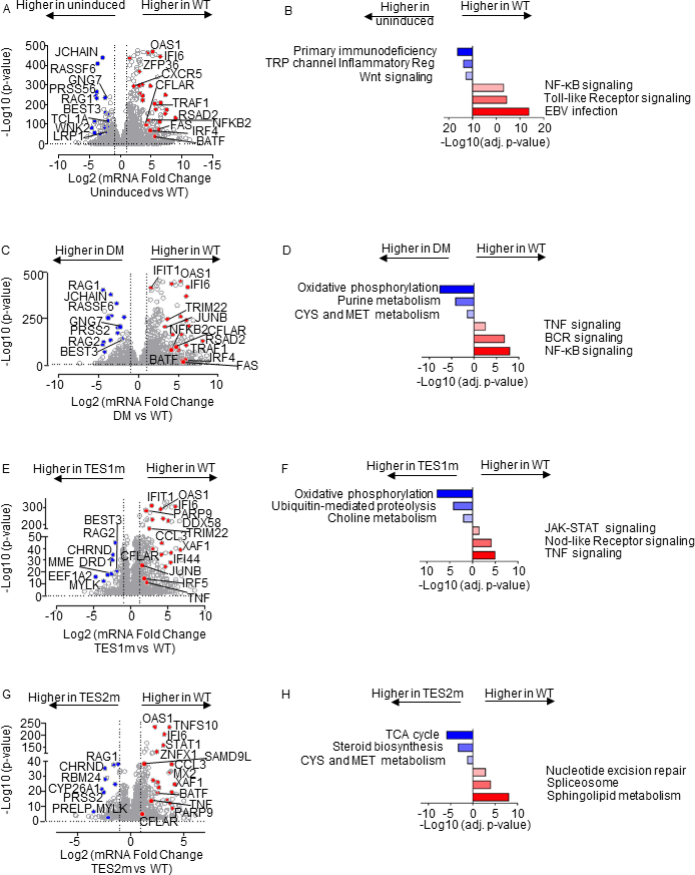
Characterization of Akata B-cell pathways targeted by TES1 vs TES2 signaling. (**A**) Volcano plot analysis of host transcriptome-wide genes differentially expressed in Akata cells conditionally induced for WT LMP1 for 24 h by 250 ng/mL Dox vs in mock-induced cells. Higher *x*-axis fold changes indicate genes more highly expressed in cells with WT LMP1 expression, whereas lower *x*-axis fold changes indicate higher expression in cells mock induced for LMP1. Data are from *n* = 3 RNAseq data sets, as in Fig. 5. (**B**) Enrichr analysis of KEGG pathways most highly enriched in RNAseq data as in panel **A** among genes more highly expressed in Akata with WT LMP1 (red) vs among genes more highly expressed with mock LMP1 induction (blue). (**C**) Volcano plot analysis of host transcriptome-wide genes differentially expressed in Akata cells conditionally induced for WT vs DM LMP1 expression for 24 h by 250 ng/mL Dox. Higher *x*-axis fold changes indicate genes more highly expressed in cells with WT LMP1 expression, whereas lower *x*-axis fold changes indicate higher expression in cells with DM LMP1. Data are from *n* = 3 RNAseq data sets, as in Fig. 5. (**D**) Enrichr analysis of KEGG pathways most highly enriched in RNAseq data as in panel **C** among genes more highly expressed in Akata with WT LMP1 (red) vs among genes more highly expressed with DM LMP1 (blue). (**E**) Volcano plot analysis of host transcriptome-wide genes differentially expressed in Akata cells conditionally induced for WT vs TES1m LMP1 for 24 h by 250 ng/mL Dox. Higher *x*-axis fold changes indicate genes more highly expressed in cells with WT LMP1, whereas lower *x*-axis fold changes indicate higher expression in cells with TES1m LMP1. Data are from *n* = 3 RNAseq data sets, as in Fig. 5. (**F**) Enrichr analysis of KEGG pathways most highly enriched in RNAseq data as in panel **E** among genes more highly expressed in Akata with WT LMP1 (red) vs among genes more highly expressed with TES1m LMP1 induction (blue). (**G**) Volcano plot analysis of host transcriptome-wide genes differentially expressed in Akata cells conditionally induced for WT vs TES2m LMP1 for 24 h by 250 ng/mL Dox. Higher *x*-axis fold changes indicate genes more highly expressed in cells with WT LMP1 expression, whereas lower *x*-axis fold changes indicate higher expression in cells with TES2m LMP1. Data are from *n* = 3 RNAseq data sets, as in Fig. 5. (**H**) Enrichr analysis of KEGG pathways most highly enriched in RNAseq data as in panel **G** among genes more highly expressed in Akata with WT LMP1 (red) vs among genes more highly expressed with TES2m LMP1 induction (blue).

We next cross-compared the most highly differentially expressed genes across the LMP1 conditions. At a fold change >2 and adjusted *P* value <0.05 cutoff, WT LMP1 highly upregulated 1,021 and downregulated 518 Akata genes, respectively. The most highly upregulated genes included multiple interferon-stimulated genes, TRAF1, FAS, and CFLAR (Fig. 6A). Interestingly, WT LMP1 decreased expression of the recombinase RAG1 and RAG2 mRNAs, as well as MME, which encodes CD10, a plasma membrane protein that we and others have found is downmodulated by EBV latency III (71–73). Enrichr analysis identified that EBV infection was the KEGG pathway most highly upregulated by WT LMP1 (Fig. 6B), reflecting the major LMP1 contribution to latency III datasets used in KEGG. Likewise, NF-κB and TLR signaling was also highly enriched, whereas primary immunodeficiency was the KEGG pathway most highly repressed by LMP1. Highly concordant effects were observed in the BL-41 cell context, where the same KEGG pathways were the most enriched among LMP1 upregulated genes (Fig. S7A through C). Cross-comparison of expression patterns in Akata and BL-41 with WT vs DM LMP1 again revealed highly concordant results (Fig. 6C and D; Fig. S7D through F), further validating a range of host genes as targets of TES1 and 2 signaling.

To gain insights into how TES2 signaling shapes LMP1 genome-wide targets, we next cross-compared transcriptomes from Akata expressing WT vs TES1 mutant LMP1. At a fold-change >2 and adjusted *P*-value < 0.05 cutoff, 561 genes were more highly expressed in WT than LMP1 TES1m, whereas 201 were less highly expressed. Interestingly, multiple interferon-stimulated genes, including IFIT1, IFI6, STAT1, and IFI44, were among the most highly upregulated in WT LMP1-expressing cells (Fig. 6E). Enrichr analysis identified TNF, Nod-like receptor (NLR) and JAK/STAT signaling to be the most highly enriched KEGG pathways among genes more highly expressed in WT LMP1+ cells, whereas oxidative phosphorylation was the most highly enriched KEGG pathway among genes more highly expressed in cells expressing the TES1 mutant (Fig. 6F). Similar analyses on BL-41 cell data sets again revealed large numbers of differentially expressed genes in WT vs TES1m LMP1 expressing cells (Fig. S8A and B; Table S5).

To then gain insights into how TES1 signaling shapes LMP1 genome-wide target gene regulation, we cross-compared Akata differentially expressed genes at 24 h post WT vs TES2 mutant LMP1 expression. At a fold change >2 and adjusted *P* value <0.05 cutoff, 275 genes were more highly expressed in Akata with WT than TES2 mutant LMP1, whereas 118 were less highly expressed. Once again, multiple interferon-stimulated genes (ISG), including STAT1, IFI6, and OAS1, were more highly expressed in WT cells. Enrichr analysis identified sphingolipid signaling and metabolism to be most highly enriched KEGG pathways among genes upregulated genes, whereas TCA cycle was the most significant KEGG pathway among genes more highly expressed with TES2 mutant LMP1 expression (Fig. 6G and H). Similar numbers of genes were differentially regulated between WT and TES2m expressing cells in the BL-41 context, where Toll-like receptor signaling was the most highly enriched term among genes more highly expressed in WT LMP1+ cells (Fig S8C and D; Table S6). These analyses are consistent with a model in which TES1 and TES2 signaling additively or synergistically upregulate ISGs. Direct cross-comparison of TES1 vs TES2 signaling in Akata and BL41 further revealed pathways selectively targeted by either (Fig. S8E through H). In the Akata environment, cells expressing TES2m more highly induced ISGs, including IFIT1, IFI6, OAS, IFI44, and DDX58 (Fig. S8E). Enrichr analysis indicated that TES1 signaling most strongly induced the Nod-like receptor (NLR), necroptosis, and chemokine signaling KEGG pathways. By contrast, TES2 signaling (from the TES1 mutant) most highly induced growth hormone and multiple amino acid metabolism KEGG pathways (Fig. S8F). In BL-41 cells, interferon-stimulated genes were not as highly induced by TES2m (Fig. S8G). Since non-canonical NF-κB activity can strongly impact B cell type I interferon pathways (74), we suspect that differences in basal NF-κB activity in BL-41 may compensate to some extent to reduce this phenotype. Instead, cell adhesion molecules and TNF signaling were most highly enriched. For instance, CFLAR was significantly more highly induced by TES1 signaling, as was OTULIN, a deubiquitinating enzyme that controls TNF/NF-κB canonical pathway. FoxO and Toll-like receptor signaling were the most highly enriched KEGG pathways induced by TES2 signaling (by the TES1 mutant) in BL-41, with FOXO signaling the most selectively induced by TES1 mutant LMP1 (Fig. S8H).

We next directly cross-compared results from our LCL and Burkitt systems. Volcano plot analysis identified host cell genes whose expression was induced by Akata WT LMP1 expression but decreased by LCL LMP1 KO, suggesting that they are bona fide LMP1 targets (Fig. S9A, blue circles and Tables S1&7). This gene set included CFLAR, TRAF1, EBI3, CCL2, CD40, consistent with prior studies (27, 50, 55, 75–78). Similarly, genes whose expression was suppressed by Akata WT LMP1 expression but induced by LCL LMP1 KO were identified as LMP1-repressed host targets (Fig. S9A, red circles and Tables S1 & 7). We similarly cross-compared data from our Akata LMP1 expression and LCL LMP1 rescue data sets. Key targets of TES1 signaling, whose expression was significantly lower in Akata with TES1 mutant than WT LMP1 and also in LCLs rescued by TES1 mutant vs WT LMP1, included CFLAR, TRAF1, NFKB2, and CCL22 (Fig. S9B;Tables S2 & 8). Likewise, key TES2 targets more highly induced by WT than by TES2 mutant in both contexts included CCL22 and EBI3, whereas CR2, which encodes the EBV B-cell receptor complement receptor 2, was, instead, more highly expressed in cells with TES2m than WT LMP1 expression, suggesting it is repressed by TES2 signaling (Fig. S9C; Tables S3 & 9). Taken together, these findings serve to validate a class of host genes as LMP1 targets in the Burkitt B-cell context, although we cannot exclude that they are regulated through secondary effects.

### LMP1 TES1 and TES2 roles in LCL dependency factor BATF and IRF4 expression

**Fig 7:**
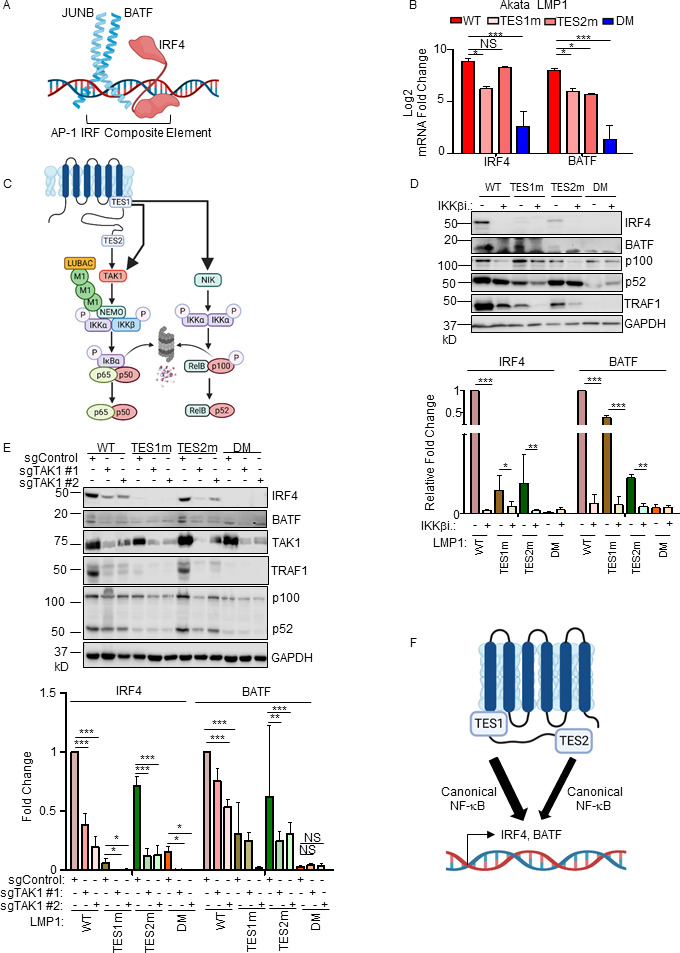
Roles of TES1 and TES2 canonical NF-κB pathways in LCL dependency factor BATF and IRF4 expression. (**A**) Schematic diagram of JUNB, BATF, and IRF4 at an AP-1/IRF composite DNA site. (**B**) Mean + SD fold changes of IRF4, BATF, and JUNB mRNA abundances from *n* = 3 RNAseq replicates of Akata cells expressing the indicated LMP1 cDNA for 24 h, as in Fig. 5. *P*-values were determined by one-sided Fisher’s exact test. *P < 0.05, ***P < 0.001. (**C**) Schematic diagram of LMP1 TES1 and TES2 NF-κB pathways. TES1 and TES2 each activate canonical NF-κB pathways, whereas TES1 also activates non-canonical NF-κB. (**D**) Immunoblot analysis of WCL from Akata cells induced for LMP1 expression by 250 ng/mL Dox for 24 h, either without or with 1 µM IKKβ inhibitor VIII. Shown below are relative fold changes + SD from *n* = 3 replicates of IRF4 or BATF vs GAPDH load control densitometry values. Values in vehicle control treated WT LMP1 expressing cells were set to 1. *P*-values were determined by one-sided Fisher’s exact test. **P < 0.001, ***P < 0.0001. (**E**) Immunoblot analysis of WCL from Cas9+ Akata cells expressing control or either of two *TAK1* targeting sgRNAs, induced for LMP1 expression by 250 ng/mL Dox for 24 h. Shown below are relative fold changes + SD from *n* = 3 replicates of IRF4 or BATF vs GAPDH load control densitometry values. Levels in cells with control sgRNA (sgControl) and WT LMP1 were set to 1. **P < 0.001, ***P < 0.0001. (**F**) Model of additive TES1 and TES2 canonical NF-κB pathway effects on BATF and IRF4 induction.

We next characterized LMP1 pathways important for BATF and IRF4 induction, given their key LCL but not Burkitt B-cell dependency factor roles (25, 79, 80). Notably, BATF and Jun family members bind cooperatively with IRF transcription factors to AP1-IRF composite DNA elements (AICE) (81), and JunB is the Jun family member predominantly expressed in LCLs (Fig. 7A). WT and TES2m LMP1 upregulated IRF4 mRNA abundance to a similar extent in Akata, whereas TES1m did so to a somewhat lesser extent. By contrast, TES1m and TES2m each upregulated BATF, but not quite as strongly as WT LMP1 (Fig. 7B). Taken together with the LCL LMP1 knockout data, these results suggest that LMP1 TES1 and TES2 signaling each support expression of BATF and IRF4.

We next examined the contribution of LMP1 NF-κB pathways to B cell IRF4 and BATF expression. LMP1 CRISPR knockout in GM12878 LCLs or in latency III Jijoye Burkitt cells significantly reduced BATF and IRF4 expression at the protein level, though effects on IRF4 were more subtle at this early timepoint (Fig. S10A). LMP1 induction of IRF4 and BATF was strongly impaired by induction of either TES1m or TES2m in Akata or in LCLs, relative to levels in cells with WT LMP1 (Fig. 7B; Fig. S10B). To test canonical NF-κB pathway roles in IRF4 and BATF induction, we next induced LMP1 in the absence or presence of a small molecule antagonist of the kinase IKKβ, which is critical for canonical NF-κB pathway signaling. IKKβ inhibition blocked their residual induction by TES1m and TES2m (Fig. 7C and D). The IKKβ inhibitor also reduced BATF and IRF4 expression in GM12878 at an early timepoint where most cells remained viable (Fig. S10C). Similar results were obtained in Akata cells that co-induced LMP1 with an IκBα super-repressor (IκBα-SR), in which IκBα serine 32 and 36 to alanine point mutations prevent its canonical pathway phosphorylation and proteasomal degradation (Fig. S10D). Furthermore, CRISPR KO of the canonical NF-κB pathway kinase TAK1 significantly impaired IRF4 induction by WT and also by TES2m LMP1 (Fig. 7E). Taken together, these results suggest that canonical NF-κB pathways driven by TES1 and also by TES2 signaling are each important for BATF and IRF4 expression (Fig. 7F).

### LMP1 TES1 and TES2 roles in EBV super-enhancer target induction

**Fig 8:**
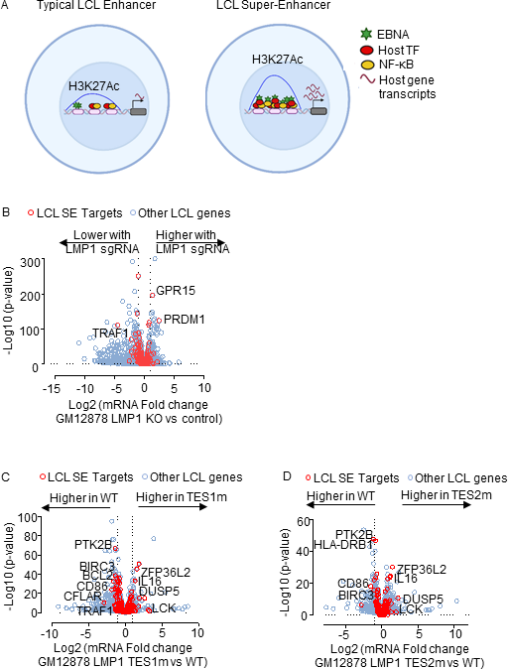
LMP1 TES1 and TES2 roles in LCL EBV super-enhancer target gene regulation. (**A**) Schematic diagram of typical LCL enhancers vs super-enhancers. Super-enhancers have significantly broader and taller histone 3 lysine 27 acetyl (H3K27Ac) peaks. EBV SE are host genomic enhancer sites bound by all five LMP1-activated NF-κB transcription factor subunits, EBNA-2, LP, 3A, and 3C. (**B**) Volcano plot analysis of mRNA values in GM12878 expressing LMP1 vs control sgRNAs as in Fig. 1. Genes targeted by EBV super-enhancers (SE) are highlighted by red circles, whereas other LCL genes are indicated by blue circles. Genes more highly expressed with LMP1 KO have higher *x*-axis values, whereas those downmodulated by LMP1 KO have lower values. *P* value <0.05 and >2-fold gene expression cutoffs were used. (**C**) Volcano plot analysis of mRNA values in GM12878 expressing LMP1 sgRNA with TES1m vs WT LMP1 cDNA rescue, as in Fig. 2 and 3. Genes targeted by EBV super-enhancers (SE) are highlighted by red circles, whereas other LCL genes are indicated by blue circles. Genes more highly expressed with endogenous LMP1 KO and TES1m rescue have higher *x*-axis values, whereas those more highly expressed with WT LMP1 rescue have lower values. *P* value <0.05 and >2-fold gene expression cutoffs were used. (**D**) Volcano plot analysis of mRNA values in GM12878 expressing LMP1 sgRNA with TES2m vs WT LMP1 cDNA rescue, as in panel **C**. Genes targeted by EBV super-enhancers (SE) are highlighted by red circles, whereas other LCL genes are indicated by blue circles. Genes more highly expressed with endogenous LMP1 KO and TES2m rescue have higher *x*-axis values, whereas those more highly expressed with WT LMP1 rescue have lower values. *P* value <0.05 and >2-fold gene expression cutoffs were used.

The five LMP1-activated NF-κB transcription factor subunits and four EBNAs target a set of LCL host genome enhancers termed EBV super-enhancers (EBV SE). EBV SE are characterized by occupancy by all five NF-κB subunits, EBNAs 2, LP, 3A, and 3C and markedly higher and broader histone H3K27ac ChIP-seq signals than at typical LCL enhancers (Fig. 8A). SE are critical for cell identity and oncogenic states (82), and EBV SE are important for LCL growth and survival (83–85). However, little has remained known about the extent to which LMP1 TES1 vs TES2 signaling contribute to EBV SE. To gain insights, we first plotted EBV SE target gene responses to GM12878 LMP1 CRISPR KO. At the early timepoint of 48 h after LMP1 editing, expression of SE target TRAF1 was significantly decreased, while expression of PRDM1 (which encodes the transcription repressor BLIMP1) and GPR15 (which encodes a G-protein coupled chemokine receptor) significantly increased. However, most other EBV SE gene targets did not significantly change at this early timepoint (Fig. 8B, red circles and Table S7). These data suggest that EBV SE are robust to short-term perturbations of LMP1 expression, perhaps given persistence of the established epigenetic landscape built at these key sites.

To then identify the extent to which LMP1, TES1 or TES2 signaling support LCL EBV SE target gene expression, we visualized effects of WT, TES1m, or TES2m rescue on EBV SE expression in LMP1 KO LCLs. CFLAR, BCL2, and TRAF1 levels were significantly lower with TES1m than with WT rescue, whereas rescue with either TES mutant significantly lowered levels of CD86 and BIRC3 messages from levels observed with WT rescue (Fig. 8C and D; Tables S8 and 9). As a complementary approach, we also analyzed effects of conditional Burkitt LMP1 expression on genes targeted by EBV SE in LCLs. In Akata B cells, WT LMP1 induction was sufficient to significantly upregulate the majority of EBV SE target gene mRNAs (Fig. S11A; Table S1). This result suggests that while EBNA-2, LP, 3A, and 3C also target these genes in LCLs, LMP1 can independently alter most of their expression, albeit not necessarily to the same extent as latency III. By contrast, TES1m or TES2m induction less strongly induced most EBV SE targets in Akata than WT LMP1 (Fig. S11B and C). A similar pattern was observed in BL-41 cells (Fig. S11D through F; Table S4). Taken together, our results suggest that TES1 and TES2 play key joint roles in the induction of genes targeted by EBV SE in LCLs.

## DISCUSSION

**Fig 9:**
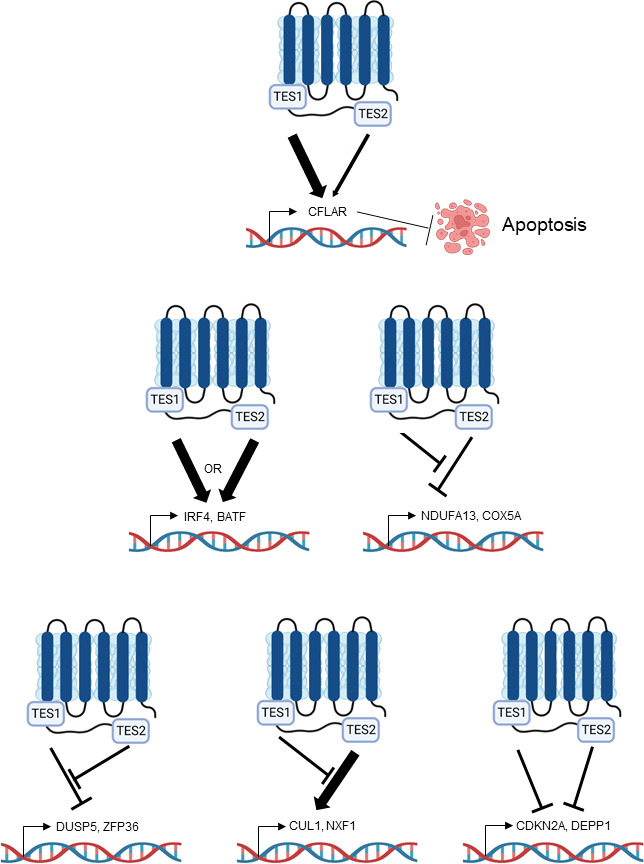
Model highlighting different modes of LMP1 TES1 and TES2 cross-talk in B-cell target gene regulation.

Why the LMP1 C-terminal tail TES1 and TES2 domains are each necessary for lymphoblastoid B-cell immortalization, and whether each is necessary for LCL survival have remained longstanding questions. To gain insights into key LMP1 B-cell roles, we used a novel LCL LMP1 KO with conditional LMP1 rescue system to identify that signaling by TES1, but not TES2, is required for LCL survival. We performed systematic B-cell transcriptome-wide analyses to identify effects of LMP1 knockout in the absence or presence of rescue by WT, TES1 mutant, or TES2 mutant LMP1, at early timepoints where cells remained viable. These highlighted key LMP1 TES1 and TES2 roles in support of LCL dependency factor and EBV super-enhancer target gene expression. As a complimentary approach to identify host B-cell genome-wide targets of LMP1 signaling, we also profiled EBV-negative Burkitt B-cell responses to conditional expression of wild-type LMP1, or LMP1 point mutants abrogated for signaling by TES1 or TES2. As has previously been described in comparisons of LMP1 vs LMP2A expression (86), TES1 and TES2 signaling effects were not simply additive, but yielded distinct effects on host target genes, with either TES domain more strongly inducing or repressing genes at particular sites, but opposing one another at other sites to fine tune target gene expression (Fig. 9).

LMP1 KO rapidly altered expression of ~3,400 LCL genes, with roughly similar numbers of host genes being downregulated as upregulated. This result is consistent with prior microarray analyses of LMP1 targets in 293 cells induced for TES2 signaling and in Burkitt-cells induced for LMP1 (27, 50), as well as in microarray analysis of EBV-infected B-cells at timepoints where LMP1 expression increases (87), further suggesting that LMP1 strongly remodels the transcriptome by pleotropic effects on host gene expression. Similar numbers of LMP1 targets were also found in microarray profiling of B-cells with transgenic LMP1 expression (77). While TES1 and TES2 induce host genes through activation of NF-κB, MAP kinase, PI3K, and interferon regulatory factor pathways (3–5, 11–20), comparatively little is known about how LMP1 downmodulates target gene expression. However, one mechanism by which LMP1 may repress target genes could be through NF-κB complexes, including p50 or p52 homodimers, or p50:52 heterodimers, potentially together with BCL3 (88, 89), as these NF-κB complexes lack transactivation domains. We do not suspect that these changes were secondary to cell death, as we performed profiling on viable cells at an early timepoint post-CRISPR editing. However, the result that LMP1 is critical for LCL survival builds on prior analyses, which showed that blockade of LMP1/NF-κB signaling triggers LCL apoptosis (50, 87, 90, 91).

Conditional expression of WT LMP1 rescued LCL survival, confirming on-target CRISPR effects on EBV genomic LMP1. Our rescue approach identified that loss of TES1, but not TES2 signaling, triggered LCL apoptosis, as judged by upregulation of caspase 3 and 7 activity and by FACS analysis for plasma membrane annexin V. Disruption of cell death signaling is a hallmark of cancer (92), and enrichment analysis identified that the KEGG apoptosis pathway was highly altered by loss of TES1. Notably, LMP1 has thus far remained an undruggable target. Therefore, these results suggest that small molecule or peptide inhibitors that block TES1 signaling may have therapeutic benefit, even in the absence of effects on TES2, for instance, in the setting of EBV-driven post-transplant and central nervous system lymphomas, which frequently express the latency III program and which are modeled by LCLs. It will be of interest to determine whether TES1 signaling has similarly important roles in apoptosis blockade in other EBV-infected tumor contexts, including in Hodgkin lymphoma Reed-Sternberg tumor cells and in nasopharyngeal carcinoma, where little is presently known about TES1 vs TES2 roles.

The LCL dependency factor CFLAR, which encodes the extrinsic apoptosis pathway inhibitor c-FLIP, was highly downmodulated upon loss of TES1 signaling, to a significantly greater extent than upon loss of TES2 signaling. This is consistent with prior microarray analysis that identified cFLIP as an LMP1 target (50), which we now identify as mostly induced by TES1 signaling. We previously identified that c-FLIP is required for LCL survival and is required to block an extrinsic apoptosis pathway that is otherwise triggered by TNFα signaling, likely in response to EBV oncogenic stress (25). Therefore, our data suggest that TES1 signaling is required for LCL survival, at least in part due to obligatory roles in cFLIP induction. Our data raise the interesting question of why CFLAR expression is particularly dependent on TES1 signaling. It is plausible that a TES1-driven non-canonical NF-κB pathway is particularly important for cFLIP transcription. However, TES1 signaling also strongly activates canonical NF-κB pathways (13, 15, 89), which may instead be critical for CFLAR induction. Alternatively, MAP kinases or PI3K activated by TES1 (4, 36, 79, 88, 93–95) may also support CFLAR expression.

LMP1 expression both activates and blocks apoptosis (42, 43, 96), and our data suggest that TES1 induction of CFLAR is central to this balance, perhaps together with BCL2 family members such as BFL1 (96, 97). However, in addition to targeting CFLAR, apoptosis pathways were enriched among LMP1 target genes. It has also been reported that the six LMP1 transmembrane domains induce apoptosis through activation of an unfolded protein response, while LMP1 C-terminal domain signaling counteracts this (96). Similarly, LMP1 induces *c-jun*, *junB,* and *junD* (98), which may play roles in balancing proliferation and apoptosis responses (99). LMP1 also closely regulates the expression of pro-apoptotic and anti-apoptotic genes to allow for cell proliferation (98). We also observed downregulation of the p53 antagonist MDM2 and upregulation of p53 upon LCL LMP1 KO. Both TES1 and TES2 had important roles in regulation of MDM2 expression.

These results further highlight LMP1 TES1 and TES2 roles in support of additional LCL dependency factors, in particular BATF and IRF4. In contrast to CFLAR, TES1 and TES2 signaling were each portant for BATF and IRF4 expression, both in LCL and in Burkitt cell models. TES1- and TES2-driven canonical NF-κB signaling supported BATF induction in EBV-negative Burkitt cells, where EBNA2 is not expressed. BATF and IRF4 expression rapidly decreased upon LMP1 KO in LCLs, even upon rescue by LMP1 signaling from only one TES domain. LMP1 canonical NF-κB pathways were also critical for inducing IRF4, which binds with BATF to composite AICE DNA sites. As EBNA2 also supports BATF (100) and EBNA3C also supports IRF4 expression (101), our results further highlight BATF and IRF4 as major hubs of EBV oncoprotein cross-talk.

We previously used ChIP-seq to characterize the LCL NF-κB genomic binding landscape (32). Rather than identifying readily recognizable LMP1 canonical vs non-canonical NF-κB target genes, this study identified complex patterns of occupancy by the five NF-κB transcription factor subunits at LCL enhancers and promoters. However, LCL enhancers often target multiple genes, often from long distances (83), complicating cross-comparison with this study. Furthermore, concurrent LMP1 TES1 and TES2 signaling yields up to 13 distinct NF-κB transcription factor dimers in LCL nuclei (32), including dimers such as cRel:p52 that are under control of both NF-κB pathways. Conditional expression of TES1m or TES2m should yield a considerably less complex NF-κB landscape. Therefore, a future objective wille be to perform NF-κB ChIP-seq using the conditional TES1m and TES2m conditional Burkitt models reported here, as these may yield less complex patterns of NF-κB occupancy.

Our analyses highlight independent, shared, and antagonistic LMP1 TES1 and TES2 roles in B-cell genome-wide target gene regulation (Fig. 9). How independent or combined TES1 and TES2 signaling have different effects on clusters of target genes will be important to define. Our results suggest multiple testable models. For instance, with regard to genes which were induced weakly by TES2 signaling, somewhat more by TES1 signaling but more highly by WT LMP1, we speculate that TES1 and TES2 may cross-talk at the epigenetic level. For example, our results are consistent with a model in which TES1 signaling increases chromatin accessibility at these sites, including the genes encoding IFIT1 and CXCL9. Once accessible, both TES1 and TES2 signal-dependent pathways may then additively or perhaps synergistically upregulate these sites. Alternatively, TES1 signaling could be needed to dismiss a repressor, such that these sites can then be stimulated by LMP1. TES1 signaling may also activate a key positive regulator such as BCL3 (88), which may then function together with transcription factors activated by TES1 and TES2 pathways. By contrast, a large number of genes appeared to be repressed by both TES1 and TES2 signaling. TES1 and TES2 may recruit co-repressors to these sites, may additively recruit the same repressor, or may reduce chromatin accessibility.

We also identified a cluster of genes repressed by unopposed TES2 signaling (Fig. 9). In Akata, this cluster (Cluster 3) was enriched for metabolism genes, raising the possibility that another key TES1 signaling role is to support metabolic pathway remodeling by EBV, such as glutathione metabolism or OXPHOS (86, 102–104). It is possible that TES2 induces a repressor that targets these sites, but that TES1 signaling serves to blunt its induction. Alternatively, TES1 signaling may induce an activator that counter-balances TES2-driven repressor activity. Or, TES2 signaling may reduce chromatin accessibility at these sites in the absence of TES1. By contrast, genes in Akata Cluster 5 were upregulated by unopposed TES2 signaling but not by WT LMP1 or unopposed TES1 signaling (Fig. 9). TES1 signaling may instead recruit repressors or alter chromatin accessibility at these sites. Epigenetic analyses of histone repressive marks, such as ChIP studies of H3K9me3 and H3K27me3, as well as ATAC-seq studies of DNA packaging, should help differentiate between these and other possibilities.

Our studies provide insights into TES1 and TES2 roles in regulation of B-cell EBV SE targets. Although EBV SE are highly co-occupied by all five LMP1-activated NF-κB subunits, individual TES1 and TES2 roles in EBV SE target gene regulation have remained unstudied. Interestingly, while either TES1 or TES2 signaling was sufficient to induce many EBV SE targets, TES1 induced a larger number, perhaps because it highly induces both canonical and non-canonical pathways and, therefore, activates all five NF-κB subunits. By contrast, LMP1 KO perturbed expression of only a small number of EBV SE targets in LCLs, likely because of the early timepoint profiled prior to cell death, which left little time for epigenetic remodeling of these sites.

WT or DM LMP1 expression caused highly concordant changes in host gene expression in Akata and BL-41 Burkitt models. We speculate that differences in response to signaling by TES1 or TES2 alone may have instead arisen from distinct host genome mutation landscapes between these two human tumor-derived models, which alter the basal NF-κB level. Nonetheless, since EBV can infect a wide range of B-cells, including of distinct differentiation or activation states that alter NF-κB states, differences between Akata and BL-41 provide insights into how LMP1 may function in differing human B-cell contexts and suggest that LMP1 may have evolved signaling by both TES domains to increase robustness across the spectrum of infected B-cell states.

LMP1 polymorphisms have been observed across EBV strains, in particular between type I and II EBV (105). In addition, LMP1 C-terminal tail polymorphisms have been observed in several analyses of EBV genomes isolated from Hodgkin lymphoma and nasopharyngeal carcinoma tumor cells. However, the roles of these variant LMP1 sequences remain controversial. Among the best studied is a 30 bp deletion present in the EBV CAO and 1510 strains, isolated from Asian NPC tumors, which causes loss of LMP1 residues 343–352 (106, 107). This 30 bp deletion has also been reported as enriched in EBV genomes isolated from Hodgkin tumor samples (107–110). A meta-analysis of 31 observational studies suggested a possible association between this LMP1 C-terminal tail deletion and nasopharyngeal carcinoma susceptibility, but was limited by small sample size and considerable variation between studies (111). Deletion of these bases, which encode the 8 residues prior to the TES2/CTAR2 domain and the first two TES2/CTAR2 residues, enhances rodent fibroblast transformation by LMP1 (112) and may reduce immunogenicity (113), but was not found to enhance LMP1-mediated NF-κB activation (114). EBV strains have multiple additional LMP1 amino acid polymorphisms which are implicated in enhanced NF-κB activation and which map to the LMP1 transmembrane domains (114). Little information is presently available about how these polymorphisms alter LMP1 target gene expression. It will therefore be of interest to use the approaches presented here to characterize how LMP1 polymorphisms, present in tumor-derived EBV strains, may alter transcriptome responses to TES1 and TES2 signaling.

In summary, we identified LMP1 genome-wide B-cell targets and characterized their responses to signaling by TES1 and/or TES2. Signaling by TES1, but not TES2, was identified to be critical for blockade of LCL apoptosis, and CFLAR was identified as the LCL dependency factor most strongly impacted by shutoff of TES1 signaling as opposed to TES2. CRISPR KO approaches highlighted LCL genes that are highly sensitive to loss of TES1 and/or TES2 signaling. *K*-means analysis highlighted gene clusters with distinct expression responses to signaling by one or both LMP1 transformation essential domains in the latency III LCL vs EBV-negative Burkitt B-cell contexts. These studies highlight multiple levels by which TES1 and TES2 signaling alter LMP1 target gene expression, including by additive vs opposing roles. Collectively, our studies provide new insights into non-redundant vs joint TES1 and TES2 roles in B-cell target gene regulation, and highlight TES1 signaling as a key lymphoblastoid B-cell therapeutic target.

## MATERIALS AND METHODS

### Cell lines, culture, and vectors

HEK293T cells were purchased from ATCC and cultured in Dulbecco’s modified Eagle medium (DMEM, Gibco) with 10% fetal bovine serum (FBS, Gibco). EBV-negative Akata and BL-41 cells were obtained from Elliott Kieff; GM12878 were purchased from Coriell. Mutu I was obtained from Jeff Sample, and Jijoye was purchased from ATCC. All B-cell lines stably expressed *Streptococcus pyogenes* Cas9 and were grown in Roswell Park Memorial Institute (RPMI) 1,640 (Life Technologies) with 10% fetal bovine serum (Gibco) and penicillin-streptomycin in a humidified chamber with 5% carbon dioxide. LMP1 wildtype, TES1 alanine point mutant 204PQQAT208 → AQQAT, TES2 384YYD386 → ID mutant and double mutant LMP1 with both AQQAT and ID mutations were cloned into the pLIX-402 vector. pLIX-402 uses a TET-On TRE promoter to drive transgene expression and a C-terminal HA-tag fusion. Lentivirus vectors were used to establish stable Cas9+/GM12878, Cas9+/EBV− Akata and Cas9+/EBV− BL-41 Burkitt cells. Cell lines were then maintained with 0.5 µg/mL puromycin or 25 µg/mL hygromycin. For LMP1 inducible expression studies, 0.5 × 10^6^ cells/mL were plated on Day 1 in 2 mL of fresh RPMI in a 12-well plate. Cell were treated with 250 ng/mL (in Burkitt cell models) for 24 h prior to sample collection for downstream analyses or with 400 ng/mL doxycycline (in LCL LMP1 cDNA rescue model) (Sigma #D9891) to allow for LMP1 rescue upon CRISPR knockout of LMP1. The Iκβα Super-repressor (SR) lacking residues 1–67 has previously been reported (27).

### Antibodies and reagents

Cell Signaling Technology (CST) TRAF1 (#4715, rabbit mAb), p105/50 (#3035, rabbit mAb), RelA (#8242, rabbit mAb), phospho-RelA (Ser536) (3033, rabbit mAb), RelB (#4922, rabbit mAb), cRel (#4727, rabbit mAb), IκBα (#9247, mouse mAb), IRF4 (#4964, rabbit mAb), BATF (#8638, rabbit mAb), TAK1 (#4505, rabbit mAb), V5 (#13202, rabbit mAb), HRP-linked anti-mouse IgG(7076), FLIP (#8510, rabbit mAb), HRP-linked anti-rabbit IgG (#7074) were used in this study at 1:1,000 dilution. p100/52 (EMD Millipore #05-361, mouse mAb, 1:1,000) and GAPDH (EMD Millipore #MAB374, mouse mAb, 1:500) were used. S12 mouse monoclonal antibody against LMP1 was purified from hybridoma supernatant (115). The IKKβ inhibitor IKK-2 inhibitor VIII (ApexBio, #A3485), puromycin dihydrochloride (Thermo Fisher #A1113803), and hygromycin B (Millipore #400052).

### Growth curve analysis

For growth curve analysis, cells were counted and then normalized to the same starting concentration, using the CellTiterGlo (CTG) luciferase assay (Promega, Cat#G7570). Live cell numbers were quantitated at each timepoint by CTG measurements, and values were corrected for tissue culture passage. Fold change of live cell number at each timepoint was calculated as a ratio of the value divided by the input value. For the Caspase-Glo 3/6 Assay (Promega #G8092), the Caspase-Glo 3/7 reagent was added to cells, mixed and incubated for 30 minutes according to the manufacturer’s instructions, followed by luminescence measurments on a Molecular Devices plate reader. Readings were normalized to respective CTG values of the samples that were performed and collected concurrently.

### CRISPR/Cas9 editing

B-cell lines with stable Cas9 expression were established as described previously (47). Briefly, HEK293T cells were plated at a density of 300,000 cells per well in 2 mL DMEM supplemented with 10% FBS on day −1. The following day (day 0) plated cells were transfected with the TransIT-LT1 Transfection Reagent (Mirus #2306) according to the manufacturer’s protocol. Transfection media were replaced by RPMI 16 hours later (day 1). B-cells were plated at 1.2 × 10^6^ density in a 6-well plate on day 1. Lentivirus collected on day 2 were added to the B-cells for spinoculation at 2,000 rpm for 2 h at 37°C and 4 µg/mL of polybrene. Spinoculated cells were placed in a humidified chamber with 5% carbon dioxide for 6 h and then pelleted and resuspended in fresh RPMI/FBS. Forty-eight hours post-transduction, transduced cells were selected by addition of puromycin 3 µg/mL or 200 µg/mL hygromycin. Broad Institute pXPR-510 control sgRNA (targets a non-coding intergenic region), Avana, or Brunello library sgRNAs, as listed in Table 1, were cloned into lentiGuide-Puro (Addgene, catalog #52963) or pLenti SpBsmBI sgRNA Hygro (Addgene, catalog #62205).

**TABLE 1:** sgRNAs used in this study

Guide no.	Gene target	sgRNA sequence (5′–3′)
#1	Control	TTGACCTTTACCGTCCCGCG
#1	LMP1	TCTATCTACAACAAAACTGG
#1	TAK1	GCTTACTGCTGGTTGCAGGG
#2	TAK1	CGCAATGAGTTGGTGTTTAC

### RNAseq

Total RNA was isolated using RNeasy mini kit (Qiagen #74106) with in-column genomic DNA digestion step (RNase-free DNase set, Qiagen #79254) according to the manufacturer’s protocol. To construct indexed libraries, 1 µg of total RNA was used for polyA mRNA selection using NEBNext Poly(A) mRNA Magnetic Isolation Module (Cat#E7490S), and library preparation with NEBNext Ultra RNA Library Prep with Sample Purification Beads (Cat#E7765S). Each experimental treatment was performed in biological triplicate. Libraries were multi-indexed (NEB 7335L and E7500S) and pooled and sequenced on an Illumina NextSeq 500 sequencer using single 75 bp read length. Adaptor-trimmed Illumina reads for each individual library were mapped back to the human GRCh37.83 transcriptome assembly using STAR2.5.2b (116). FeatureCounts was used to estimate the number of reads mapped to each contig (117). Only transcripts with at least five cumulative mapping counts were used in this analysis. DESeq2 was used to evaluate differential expression (DE) (118). DESeq2 uses a negative binomial distribution to account for overdispersion in transcriptome data sets. It is conservative and uses a heuristic approach to detect outliers while avoiding false positives. Each DE analysis was composed of a pairwise comparison between experimental group and the control group. Differentially expressed genes were identified after a correction for false discovery rate (FDR). For more stringent analyses, we set the cutoff for truly differentially expressed genes as adjusted *P* value (FDR corrected) <0.05 and absolute fold change >2. DE genes meeting this cutoff were selected and subject to downstream bioinformatics and functional analyses, including clustering, data visualization, GO annotation, and pathway analysis. DE genes were also subjected to Enrichr analysis (https://maayanlab.cloud/Enrichr/) for pathway analysis. Heatmaps were generated by feeding the Variance-Stabilizing Transformed values of selected DE genes from DESeq2 into Morpheus (https://software.broadinstitute.org/morpheus/).

### Quantitative real-time qRT-PCR analysis

Total RNA was isolated using RNeasy mini kit (Qiagen #74106) with in-column genomic DNA digestion step (RNase-free DNase set, Qiagen #79254) according to the manufacturer’s protocol. Reverse transcription was performed with 400 ng of total RNA using iScript Reverse Transcription supermix (Bio-Rad #1708841) in a 20 µL reaction. The cDNA mixture was diluted 1:20, and 4 µL of the diluted cDNA was taken to perform qPCR using the Power SYBR green PCR master mix (Fisher Scientific #4368708) in CFX96 Touch Real-Time PCR Detection System (Bio-Rad). Data were normalized to internal control 18 s RNA levels. Relative expression was calculated using 2^−ΔΔ*Ct*^ method. All samples were run in technical triplicates, and at least three independent experiments were performed. Primer sequences are outlined in Table 2.

**TABLE 2:** RT-PCR primers used in this study

Gene name	Primer sequence (5′–3′)	Primer sequence (3′–5′)
CCL22	CGCGTGGTGAAACACTTCTA	GGATCGGCACAGATCTCCT
EBI3	GATCCGTTACAAGCGTCAGG	ACGTAGTACCTGGCTCGGG
IRF4	ACAGCAGTTCTTGTCAGAG	GAGGTTCTACGTGAGCTG
18 s	CCTGCGGCTTAATTTGACTC	AACCAGACAAATCGCTCCAC

### Immunoblot analysis

Cells were lysed in Laemmeli buffer (0.2 M Tris-HCL, 0.4 M dithiothreitol, 277 mM SDS, 6 mM bromophenol blue, and 10% [vol/vol] glycerol) and sonicated at 4°C for 5 s using a probe sonicator at 20% amplitude and boiled at 95°C for 8 min. The whole cell lysates were resolved by 12% or 15% SDS-PAGE, transferred to nitrocellulose filters at 100 V at 4°C for 1.5 h, blocked with 5% non-fat dried milk in 1× TBST for 1 h at room temperature, and then probed with the indicated primary antibodies (diluted in 1 × TBS-T with 0.02% sodium azide) overnight at 4°C on a rotating platform. Blots were washed three times in TBST for 10 min each and then probed with horseradish peroxidase (HRP)-conjugated secondary antibodies at a dilution of 1:3,000 in 1× TBST with 5% non-fat dried milk for 1 h at room temperature. Blots were then washed three times in TBST for 10 min each, developed by ECL chemiluminescent substrate (Thermo Scientific, #34578), and imaged on Li-COR Odyssey workstation.

### Flow cytometry analysis

FACS was performed using a FACSCalibur instrument (BD). For ICAM-1 and Fas detection, cells were washed in PBS w/2% FBS, stained on ice for 30 min with BioLegend PE-conjugated anti-CD54/ICAM-1 and APC-conjugated anti-CD95/Fas antibody, washed three times with PBS with 2% FBS, and analyzed by FACS. 7-AAD viability assays were carried out using 7-AAD (Thermo Fisher, #A1310) where cells were harvested and washed twice in 1× PBS supplemented with 2% FBS (Gibco). Washed cells were incubated with 1 µg/mL 7-AAD solution in 1× PBS/2% FBS buffer for 5 min at room temperature and protected from light. Stained cells were analyzed via flow cytometry. Annexin V assay was performed as follows: 1 × 10^6^ cells were washed with 1× PBS twice to remove excess RPMI. Cells (2 × 10^5^) were then resuspended and stained with 5 µL of annexin V-FITC (#640945, Biolegend) in 100 uL of annexin V binding buffer (10 mM HEPES, 140 mM NaCl, and 2.5 mM CaCl_2_). Cells were incubated at room temperature for 15 min and protected from light before analyzed by FACS.

### Bioinformatic analysis and software

All the growth curves and column charts were made with GraphPad Prism v.9. FACS data were analyzed by FlowJo V10.

## Data Availability

All RNAseq and proteomics data sets are available in the GEO omnibus under accession numbers GSE228158, GSE228167, GSE228178, and GSE240732.

## SUPPLEMENTAL MATERIAL

Supplemental Figures mbio.02338-23-s0001.pdf Figures S1 to S11.

Table S1 mbio.02338-23-s0002.xlsx Log2 fold changes of gene expression in EBV-negative Akata B cells expressing LMP1 WT for 24 hours versus uninduced controls.

Table S2 mbio.02338-23-s0003.xlsx Log2 fold changes of gene expression in EBV-negative Akata B cells expressing TES1m versus WT LMP1 for 24 hours.

Table S3 mbio.02338-23-s0004.xlsx Log2 fold changes of gene expression in EBV-negative Akata B cells expressing TES2m versus WT LMP1 for 24 hours.

Table S4 mbio.02338-23-s0005.xlsx Log2 fold changes of gene expression in EBV-negative BL-41 B cells expressing LMP1 WT for 24 hours versus uninduced controls.

Table S5 mbio.02338-23-s0006.xlsx Log2 fold changes of gene expression in EBV-negative BL-41 B cells expressing TES1m versus WT LMP1 for 24 hours.

Table S6 mbio.02338-23-s0007.xlsx Log2 fold changes of gene expression in EBV-negative BL-41 B cells expressing TES2m versus WT LMP1 for 24 hours..

Table S7 mbio.02338-23-s0008.xlsx Log2 fold changes of gene expression in GM12878 expressing LMP1 targeting vs control sgRNAs.

Table S8 mbio.02338-23-s0009.xlsx Log2 fold changes of gene expression in LMP1 KO GM12878 LCLs with TES1m vs WT LMP1 cDNA rescue.

Table S9 mbio.02338-23-s0010.xlsx Log2 fold changes of gene expression in LMP1 KO GM12878 LCLs with TES2m vs WT LMP1 cDNA rescue.

ASM does not own the copyrights to Supplemental Material that may be linked to, or accessed through, an article. The authors have granted ASM a non-exclusive, world-wide license to publish the Supplemental Material files. Please contact the corresponding author directly for reuse.
