# Supplementary material for: Characterization of target gene regulation by the two Epstein-Barr virus oncogene LMP1 domains essential for B-cell transformation
Source: mBio. 2023 Nov 27;14(6):e02338-23. doi: 10.1128/mbio.02338-23 (PMC10746160; doi:10.1128/mbio.02338-23)
Supplement: Supplemental Figures — Figures S1 to S11. [file mbio.02338-23-s0001.pdf]

Figure S1

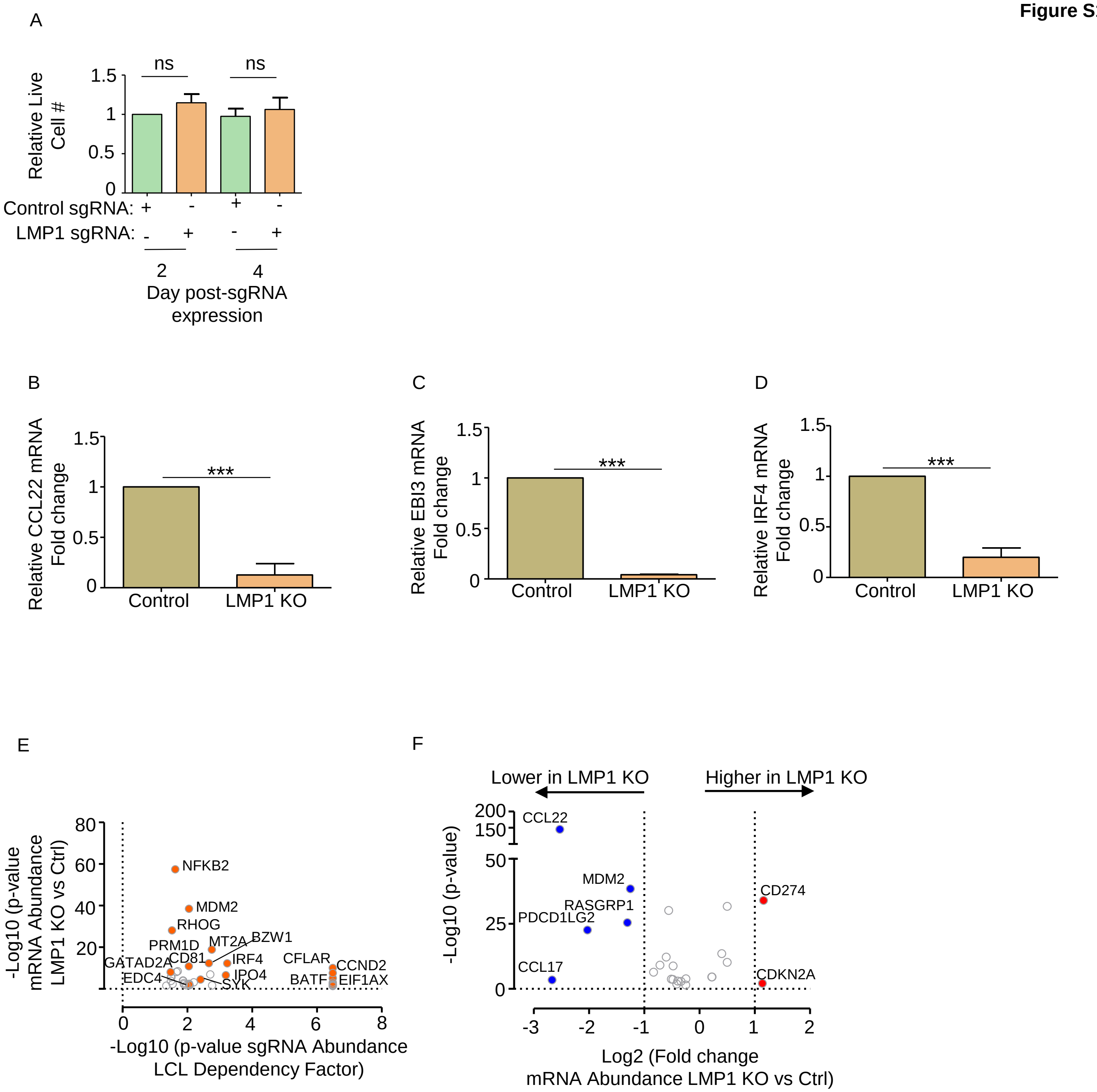

A
Relative Live Cell #
ns
ns
Control sgRNA:
LMP1 sgRNA:
Day post-sgRNA expression
B
Relative CCL22 mRNA Fold change
***
Control
LMP1 KO
C
Relative EBI3 mRNA Fold change
***
Control
LMP1 KO
D
Relative IRF4 mRNA Fold change
***
Control
LMP1 KO
E
-Log10 (p-value mRNA Abundance LMP1 KO vs Ctrl)
-Log10 (p-value sgRNA Abundance LCL Dependency Factor)
NFKB2
MDM2
RHOG
PRM1D
MT2A
BZW1
GATAD2A
CD81
IRF4
CFLAR
CCND2
EDC4
IPO4
BATF
EIF1AX
SYK
F
Lower in LMP1 KO
Higher in LMP1 KO
-Log10 (p-value)
Log2 (Fold change mRNA Abundance LMP1 KO vs Ctrl)
CCL22
MDM2
RASGRP1
PDCD1LG2
CCL17
CD274
CDKN2A

Fig. S1. Characterization of LMP1 KO effects on GM12878 LCL target gene regulation.

(A)Relative mean + standard deviation (SD) live cell numbers from CellTitreGlo analysis of n=3 replicates of Cas9+ GM12878 LCLs, transduced with lentiviruses that expressed control or LMP1 sgRNAs and puromycin selected, for 0 versus 2 days.

(B) RT-PCR analysis of CCL22 mRNA abundance in Cas9+ GM12878 post-transduction with lentiviruses that expressed control or LMP1 sgRNA and puromycin selected for 2 days, as in **Fig. 1B**. Values from cells with control sgRNA were set to 1, and mean fold-change of CCL22 mRNA abundance + SD in cells with LMP1 sgRNA are shown. p-values were determined by one-sided Fisher's exact test from two independent experiments, each with two technical replicates. ***p<0.001. The same RNA used for RNA-seq (**Fig. 1B**) was used for these qPCR experiments.

(C) RT-PCR analysis of EBI3 mRNA abundance in Cas9+ GM12878 post-transduction with lentiviruses that expressed control or LMP1 sgRNA and puromycin selected for 2 days, as in **Fig. 1B**. Values from cells with control sgRNA were set to 1, and mean fold-change of EBI3 mRNA abundance + SD in cells with LMP1 sgRNA are shown. p-values were determined by one-sided Fisher's exact test from two independent experiments, each with two technical replicates. ***p<0.001. The same RNA used for RNA-seq (**Fig. 1B**) was used for these qPCR experiments.

(D) RT-PCR analysis of IRF4 mRNA abundance in Cas9+ GM12878 post-transduction with lentiviruses that expressed control or LMP1 sgRNA and puromycin selected for 2 days, as in **Fig. 1B**. Values from cells with control sgRNA were set to 1, and mean fold-change of IRF4 mRNA abundance + SD in cells with LMP1 sgRNA are shown. p-values were determined by one-sided Fisher's exact test from two independent experiments, each with two technical replicates. ***p<0.001. The same RNA used for RNA-seq (**Fig. 1B**) was used for these qPCR experiments.

(E) Scatter plot analysis cross-comparing the significance of changes in LCL dependency factor expression upon GM127878 LMP1 KO versus the CRISPR screen significance score for selection against sgRNAs in LCL vs Burkitt dependency factor analysis (25). Shown on the Y-axis are -log10 transformed P-values from RNAseq analysis of GM12878 LCLs transduced with lentiviruses expressing LMP1 versus control sgRNA (as in **Fig. 1F**), versus -log10 transformed P-values from CRISPR LCL vs Burkitt cell dependency factor analysis (25). Higher Y-axis scores indicate more significant differences in expression for the indicated genes in GM12878 with LMP1 vs control sgRNA. Higher X-axis scores indicate a stronger selection against sgRNA targeting the indicated genes in GM12878 LCLs versus P3HR1 Burkitt cells over 21 days of cell culture. Shown are genes with p<0.05 in both analyses.

(F) Volcano plot analysis visualizing KEGG Hodgkin lymphoma pathway gene -Log10 (P-value) on the y-axis versus Log2 transformed fold change in mRNA abundances on the x-axis of GM12878 genes in cells expressing LMP1 versus control sgRNA (as in **Fig. 1F**). P-value <0.05 and >2-fold change mRNA abundance cutoffs were used.

A

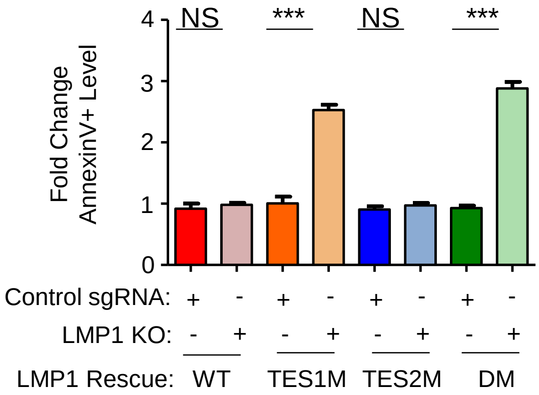
NS
***
NS
***
Fold Change
AnnexinV+ Level
0
1
2
3
4
Control sgRNA: + - + - + - + -
LMP1 KO: - + - + - + - +
LMP1 Rescue: WT TES1M TES2M DM

Fig. S2. Loss of TES1 but not TES2 signaling triggers LCL apoptosis.

Mean ± SD of fold change plasma membrane annexin V values from n=3 independent experiments, using GM12878 with the indicated control or LMP1 sgRNA and rescue cDNA expression. Values in GM12878 with control sgRNA and no LMP1 rescue cDNA were set to 1.

Figure S3

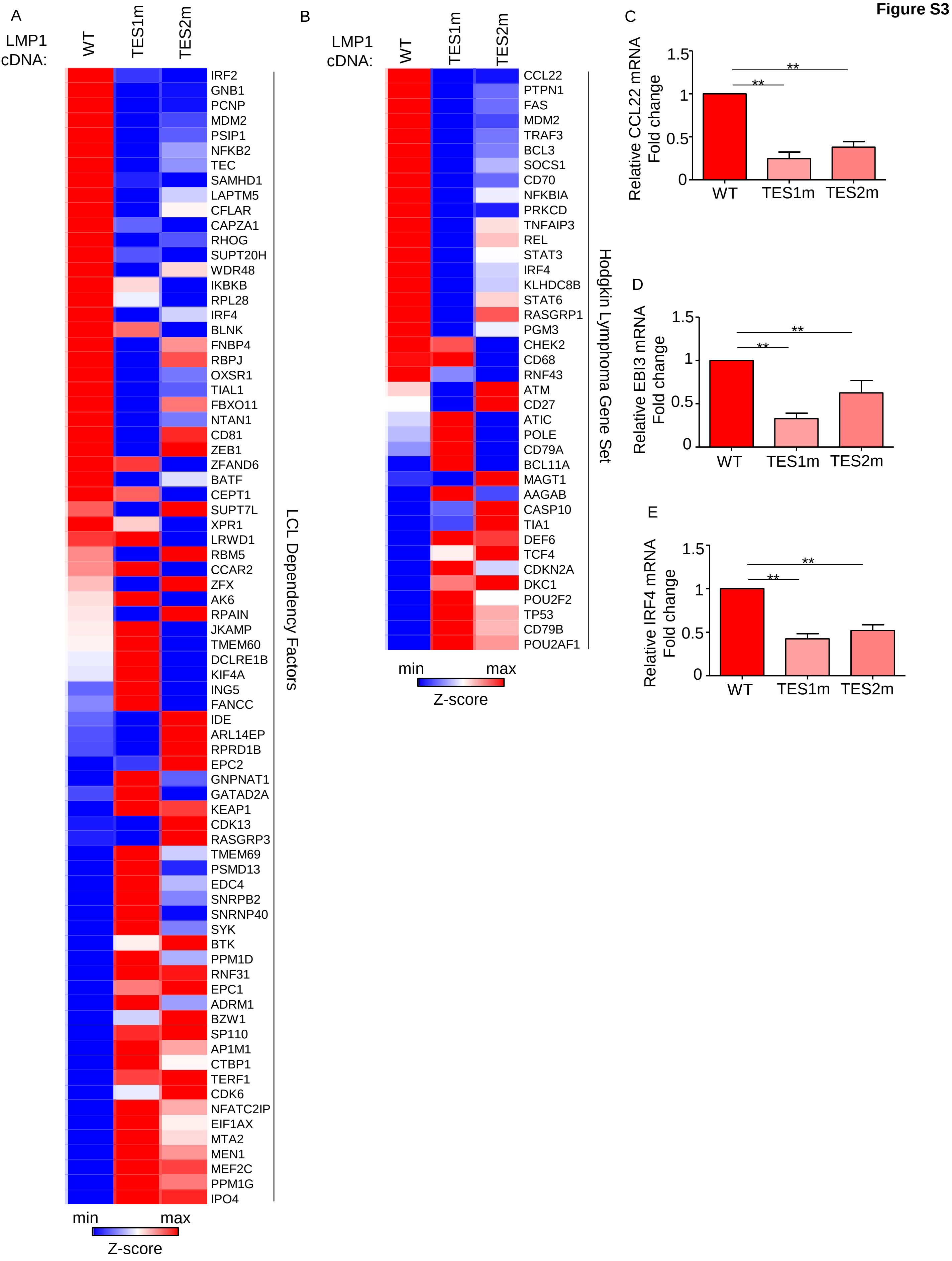
A
LMP1 cDNA:
WT
TES1m
TES2m
IRF2
GNB1
PCNP
MDM2
PSIP1
NFKB2
TEC
SAMHD1
LAPTM5
CFLAR
CAPZA1
RHOG
SUPT20H
WDR48
IKBKB
RPL28
IRF4
BLNK
FNBP4
RBPJ
OXSR1
TIAL1
FBXO11
NTAN1
CD81
ZEB1
ZFAND6
BATF
CEPT1
SUPT7L
XPR1
LRWD1
RBM5
CCAR2
ZFX
AK6
RPAIN
JKAMP
TMEM60
DCLRE1B
KIF4A
ING5
FANCC
IDE
ARL14EP
RPRD1B
EPC2
GNPNAT1
GATAD2A
KEAP1
CDK13
RASGRP3
TMEM69
PSMD13
EDC4
SNRPB2
SNRNP40
SYK
BTK
PPM1D
RNF31
EPC1
ADRM1
BZW1
SP110
AP1M1
CTBP1
TERF1
CDK6
NFATC2IP
EIF1AX
MTA2
MEN1
MEF2C
PPM1G
IPO4
LCL Dependency Factors
min
max
Z-score
B
LMP1 cDNA:
WT
TES1m
TES2m
CCL22
PTPN1
FAS
MDM2
TRAF3
BCL3
SOCS1
CD70
NFKBIA
PRKCD
TNFAIP3
REL
STAT3
IRF4
KLHDC8B
STAT6
RASGRP1
PGM3
CHEK2
CD68
RNF43
ATM
CD27
ATIC
POLE
CD79A
BCL11A
MAGT1
AAGAB
CASP10
TIA1
DEF6
TCF4
CDKN2A
DKC1
POU2F2
TP53
CD79B
POU2AF1
Hodgkin Lymphoma Gene Set
min
max
Z-score
C
Relative CCL22 mRNA Fold change
1.5
1
0.5
0
**
**
WT
TES1m
TES2m
D
Relative EBI3 mRNA Fold change
1.5
1
0.5
0
**
**
WT
TES1m
TES2m
E
Relative IRF4 mRNA Fold change
1.5
1
0.5
0
**
**
WT
TES1m
TES2m

Figure S3. Characterization of TES1 vs TES2 LCL dependency factor and Hodgkin lymphoma pathway targets.

(A) Heatmap analysis of CRISPR defined LCL dependency factor gene relative row Z-scores from RNAseq of GM12878 expressing LMP1 sgRNA and the indicated rescue cDNA, as in **Fig. 3**. The Z-score scale is shown at bottom, where blue and red colors indicate lower versus higher relative expression, respectively. Two-way ANOVA P-value cutoff of <0.05 and >2-fold gene expression cutoffs were used.

(B) Heatmap analysis of KEGG Hodgkin Lymphoma pathway gene relative row Z-scores from RNAseq of GM12878 expressing LMP1 sgRNA and the indicated rescue cDNA, as in **Fig. 3**. Two-way ANOVA P-value cutoff of <0.05 and >2-fold gene expression cutoffs were used.

(C) RT-PCR analysis of CCL22 mRNA abundance in GM12878 LCLs transduced with lentivirus expressing LMP1 sgRNA and induced for WT, TES1m or TES2m rescue cDNA expression for 6 days, as in Fig. 3A. The same RNA used for RNA-seq in Fig. 3A was used for these qPCR experiments. Values from cells with WT LMP1 rescue cDNA were set to 1, and mean fold-change of CCL22 mRNA abundance + SD in cells with TES1m or TES2m rescue cDNA are shown. p-values were determined by one-sided Fisher's exact test from two independent experiments, each with two technical replicates. ***p<0.001.

(D) RT-PCR analysis of EBI3 mRNA abundance in GM12878 LCLs transduced with lentivirus expressing LMP1 sgRNA and induced for WT, TES1m or TES2m rescue cDNA expression for 6 days, as in Fig. 3A. The same RNA used for RNA-seq in Fig. 3A was used for these qPCR experiments. Values from cells with WT LMP1 rescue cDNA were set to 1, and mean fold-change of EBI3 mRNA abundance + SD in cells with TES1m or TES2m rescue cDNA are shown. p-values were determined by one-sided Fisher's exact test from two independent experiments, each with two technical replicates. ***p<0.001.

(E) RT-PCR analysis of IRF4 mRNA abundance in GM12878 LCLs transduced with lentivirus expressing LMP1 sgRNA and induced for WT, TES1m or TES2m rescue cDNA expression for 6 days, as in **Fig. 3A**. The same RNA used for RNA-seq in Fig. 3A was used for these qPCR experiments. Values from cells with WT LMP1 rescue cDNA were set to 1, and mean fold-change of IRF4 mRNA abundance + SD in cells with TES1m or TES2m rescue cDNA are shown. p-values were determined by one-sided Fisher's exact test from two independent experiments, each with two technical replicates. ***p<0.001.

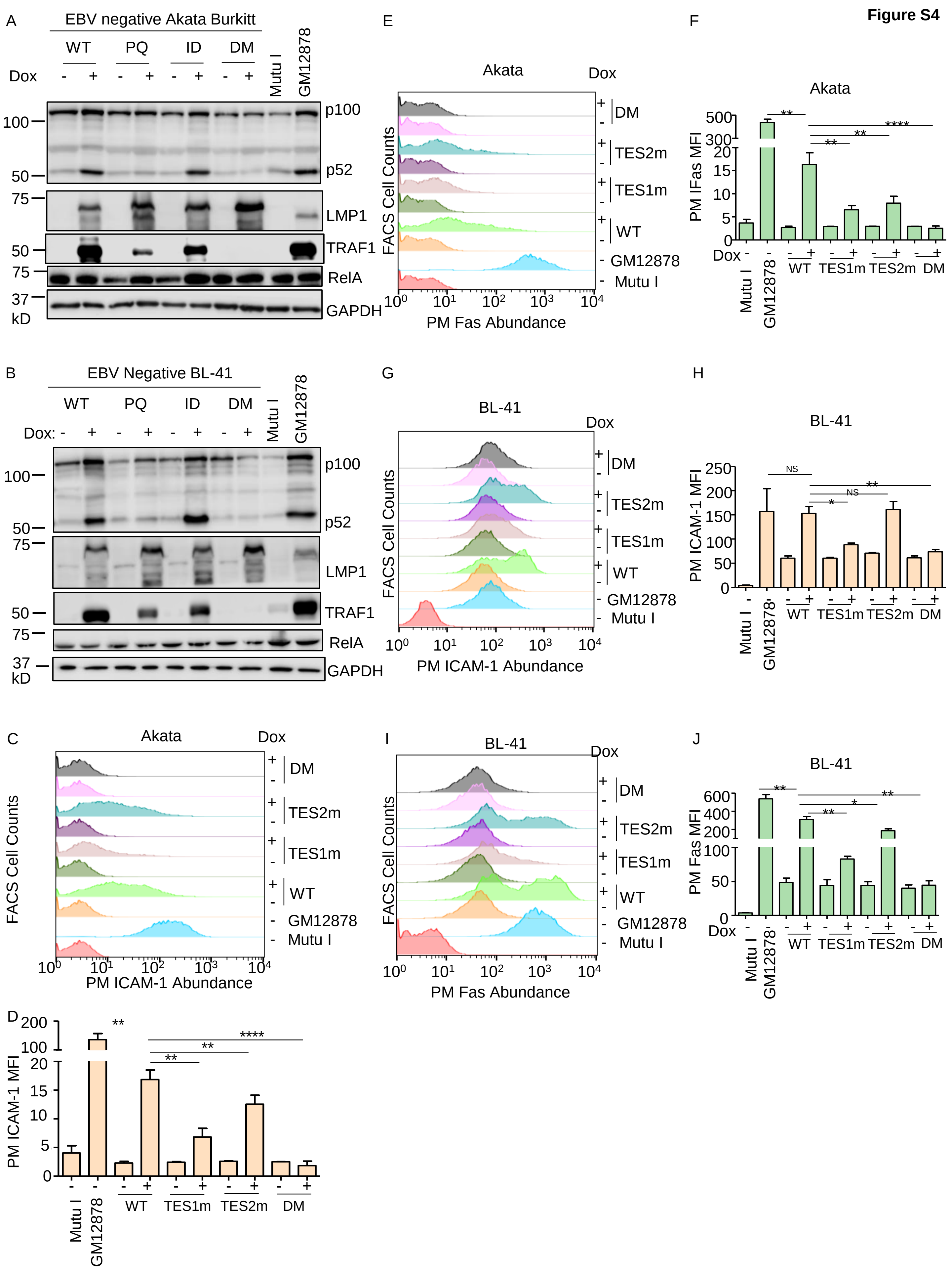
Figure S4
A
EBV negative Akata Burkitt
WT PQ ID DM Mutu I GM12878
Dox - + - + - + - +
p100
p52
LMP1
TRAF1
RelA
GAPDH
100 50 75 50 75 37 kD
B
EBV Negative BL-41
WT PQ ID DM Mutu I GM12878
Dox: - + - + - + - +
p100
p52
LMP1
TRAF1
RelA
GAPDH
100 50 75 50 75 37 kD
C
Akata
Dox
DM TES2m TES1m WT GM12878 Mutu I
FACS Cell Counts
PM ICAM-1 Abundance
D
PM ICAM-1 MFI
Mutu I GM12878 WT TES1m TES2m DM
E
Akata
Dox
DM TES2m TES1m WT GM12878 Mutu I
FACS Cell Counts
PM Fas Abundance
F
Akata
PM IFas MFI
Dox
Mutu I GM12878 WT TES1m TES2m DM
G
BL-41
Dox
DM TES2m TES1m WT GM12878 Mutu I
FACS Cell Counts
PM ICAM-1 Abundance
H
BL-41
PM ICAM-1 MFI
NS
Dox
Mutu I GM12878 WT TES1m TES2m DM
I
BL-41
Dox
DM TES2m TES1m WT GM12878 Mutu I
FACS Cell Counts
PM Fas Abundance
J
BL-41
PM Fas MFI
Dox
Mutu I GM12878 WT TES1m TES2m DM

Fig. S4. Validation of LMP1 WT, TES1m, TES2m and DM conditional expression system in EBV-negative Akata Burkitt B-cells.

(A) Immunoblot analysis of WCL from Akata cells induced for LMP1 WT, TES1m, TES2m or DM expression by addition of 250 ng/ml doxycycline (Dox) for 24 hours, as indicated. For cross-comparison, WCL from equal numbers of Mutu I Burkitt lymphoma (latency I, lacks LMP1 expression) and GM12878 were also included at right. Blots are representative of n = 3 experiments.

(B) Immunoblot analysis of WCL from BL-41 cells induced for WT, TES1m, TES2m or DM LMP1 by addition of 250 ng/ml Dox for 24 hours, as indicated. For cross-comparison, WCL from equal numbers of Mutu I Burkitt and GM12878 were also included at right. Blots are representative of n = 3 experiments.

(C) FACS analysis of plasma membrane (PM) ICAM-1 abundance in Akata cells induced for LMP1 by 250 ng/ml Dox for 24 hours, as indicated. Y-axis are histogram cell counts, X-axis represents PM ICAM-1 abundance. For comparison, levels in GM12878 LCLs or latency I Mutu I Burkitt cells are shown.

(D) PM ICAM-1 Mean Fluorescence Intensity (MFI) + standard deviation (SD) from n=3 replicates in Akata cells with the indicated LMP1 expression, as in (C). p-values were determined by one-sided Fisher's exact test. **$p<0.001$, ***p<0001.

(E) FACS analysis of PM Fas abundance in Akata cells induced for LMP1 by 250ng/ml of Dox for 24 hours as indicated. For comparison, GM12878 LCLs or latency I Mutu I Burkitt cells were also analyzed.

(F) PM Fas MFI + SD from n=3 replicates in Akata cells with the indicated LMP1 expression, as in (E). P-values were determined by one-sided Fisher's exact test. **$p<0.001$, ***$p<0.0001$.

(G) FACS analysis of PM ICAM-1 in BL-41 cells induced for LMP1 expression by 250ng/ml Dox for 24 hours, as indicated. For comparison, GM12878 and Mutu I were also analyzed. p-values were determined by one-sided Fisher's exact test. **$p<0.001$, ***$p<0.0001$.

(H) PM ICAM-1 MFI + SD from n=3 replicates in BL-41 cells with the indicated LMP1 expression, as in (G). P-values were determined by one-sided Fisher's exact test. **$p<0.001$, ***$p<0.0001$.

(I) FACS analysis of PM Fas levels in BL-41 cells induced for LMP1 expression by 250 ng/ml dox for 24 hours, as indicated. GM12878 and Mutu I were analyzed for cross-comparison. p-values were determined by one-sided Fisher's exact test. **$p<0.001$, ***$p<0.0001$.

(J) PM Fas MFI + SD from n=3 replicates of BL-41-LMP1 with the indicated LMP1 expression, as in (I). Mutu I and GM12878 were analyzed for comparison. p-values were determined by one-sided Fisher's exact test. **$p<0.001$, ***$p<0.0001$.

Figure S5

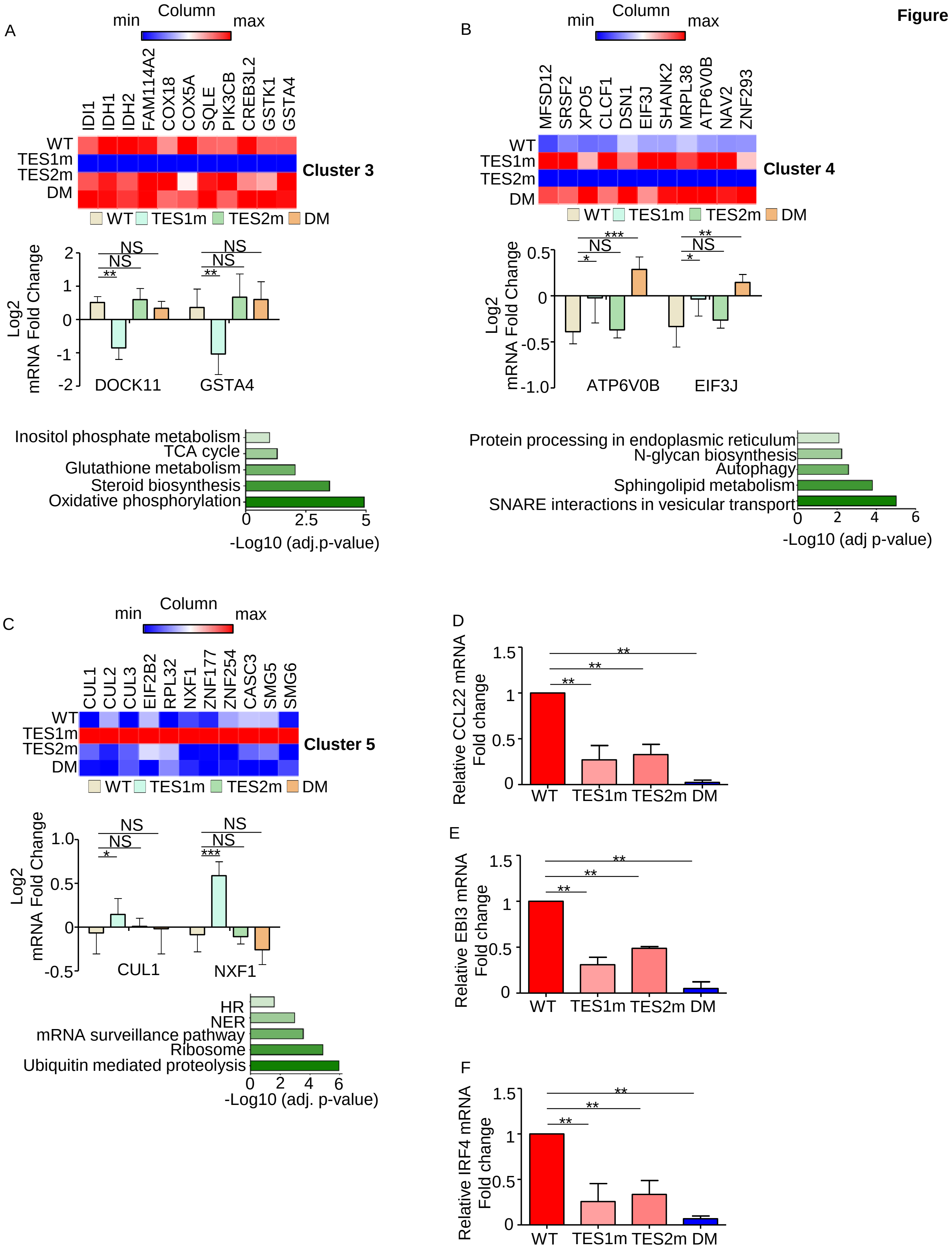
A
Column
min
max
IDI1
IDH1
IDH2
FAM114A2
COX18
COX5A
SQLE
PIK3CB
CREB3L2
GSTK1
GSTA4
WT
TES1m
TES2m
DM
Cluster 3
WT TES1m TES2m DM
Log2 mRNA Fold Change
NS
NS
**
DOCK11
GSTA4
Inositol phosphate metabolism
TCA cycle
Glutathione metabolism
Steroid biosynthesis
Oxidative phosphorylation
-Log10 (adj.p-value)
B
MFSD12
SRSF2
XPO5
CLCF1
DSN1
EIF3J
SHANK2
MRPL38
ATP6V0B
NAV2
ZNF293
Cluster 4
***
*
ATP6V0B
EIF3J
Protein processing in endoplasmic reticulum
N-glycan biosynthesis
Autophagy
Sphingolipid metabolism
SNARE interactions in vesicular transport
-Log10 (adj p-value)
C
CUL1
CUL2
CUL3
EIF2B2
RPL32
NXF1
ZNF177
ZNF254
CASC3
SMG5
SMG6
Cluster 5
CUL1
NXF1
HR
NER
mRNA surveillance pathway
Ribosome
Ubiquitin mediated proteolysis
-Log10 (adj. p-value)
D
Relative CCL22 mRNA Fold change
WT TES1m TES2m DM
E
Relative EBI3 mRNA Fold change
F
Relative IRF4 mRNA Fold change

Fig. S5. Characterization of host genome-wide Akata B-cell LMP1 target genes, related to Figure 5.

(A) Heatmaps of representative **Figure 5** Cluster 3 differentially regulated genes (top), with column maximum colored red and minimum colored blue, as shown by the scalebar. Also shown are expression values of two representative Clusters 3 genes (lower left) and Enrichr analysis of KEGG pathways most significantly enriched Cluster 3 gene sets (lower right). p-values were determined by one-sided Fisher's exact test. **p<0.01.

(B) Heatmaps of representative **Figure 5** Cluster 4 differentially regulated genes (top), with column maximum colored red and minimum colored blue, as shown by the scalebar. Also shown are expression values of two representative Clusters 4 genes (lower left) and Enrichr analysis of KEGG pathways most significantly enriched Cluster 4 gene sets (lower right). p-values were determined by one-sided Fisher's exact test. *p<0.05, **p<0.01, ***p<0.001.

(C) Heatmaps of representative **Figure 5** Cluster 5 differentially regulated genes (top), with column maximum colored red and minimum colored blue, as shown by the scalebar. Also shown are expression values of two representative Clusters 5 genes (lower left) and Enrichr analysis of KEGG pathways most significantly enriched Cluster 5 gene sets (lower right). p-values were determined by one-sided Fisher's exact test. *p<0.05, ***p<0.001.

(D) RT-PCR analysis of CCL22 mRNA abundance in cells with conditional LMP1 WT, TES1m, TES2m or DM expression induced by 250 ng/ml doxycycline for 24 hours as in Fig. 5A. The same RNA used for RNA-seq in Fig. 5A was used for these qPCR experiments. Values from cells with conditional WT LMP1 expression were set to 1, and mean fold-change of CCL22 mRNA abundance + SD in cells with conditional TES1m,TES2m or DM expression are shown. p-values were determined by one-sided Fisher's exact test from two independent experiments, each with two technical replicates. **p<0.01.

(E) RT-PCR analysis of EBI3 mRNA abundance in cells with conditional LMP1 WT, TES1m, TES2m or DM expression induced by 250 ng/ml doxycycline for 24 hours as in Fig. 5A. The same RNA used for RNA-seq in Fig. 5A was used for these qPCR experiments. Values from cells with conditional WT LMP1 expression were set to 1, and mean fold-change of EBI3 mRNA abundance + SD in cells with conditional TES1m,TES2m or DM expression are shown. p-values were determined by one-sided Fisher's exact test from two independent experiments, each with two technical replicates. **p<0.01.

(F) RT-PCR analysis of IRF4 mRNA abundance in cells with conditional LMP1 WT, TES1m, TES2m or DM expression induced by 250 ng/ml doxycycline for 24 hours as in Fig. 5A The same RNA used for RNA-seq in Fig. 5A was used for these qPCR experiments. Values from cells with conditional WT LMP1 expression were set to 1, and mean fold-change of IRF4 mRNA abundance + SD in cells with conditional TES1m,TES2m or DM expression are shown. p-values were determined by one-sided Fisher's exact test from two independent experiments, each with two technical replicates. **p<0.01.

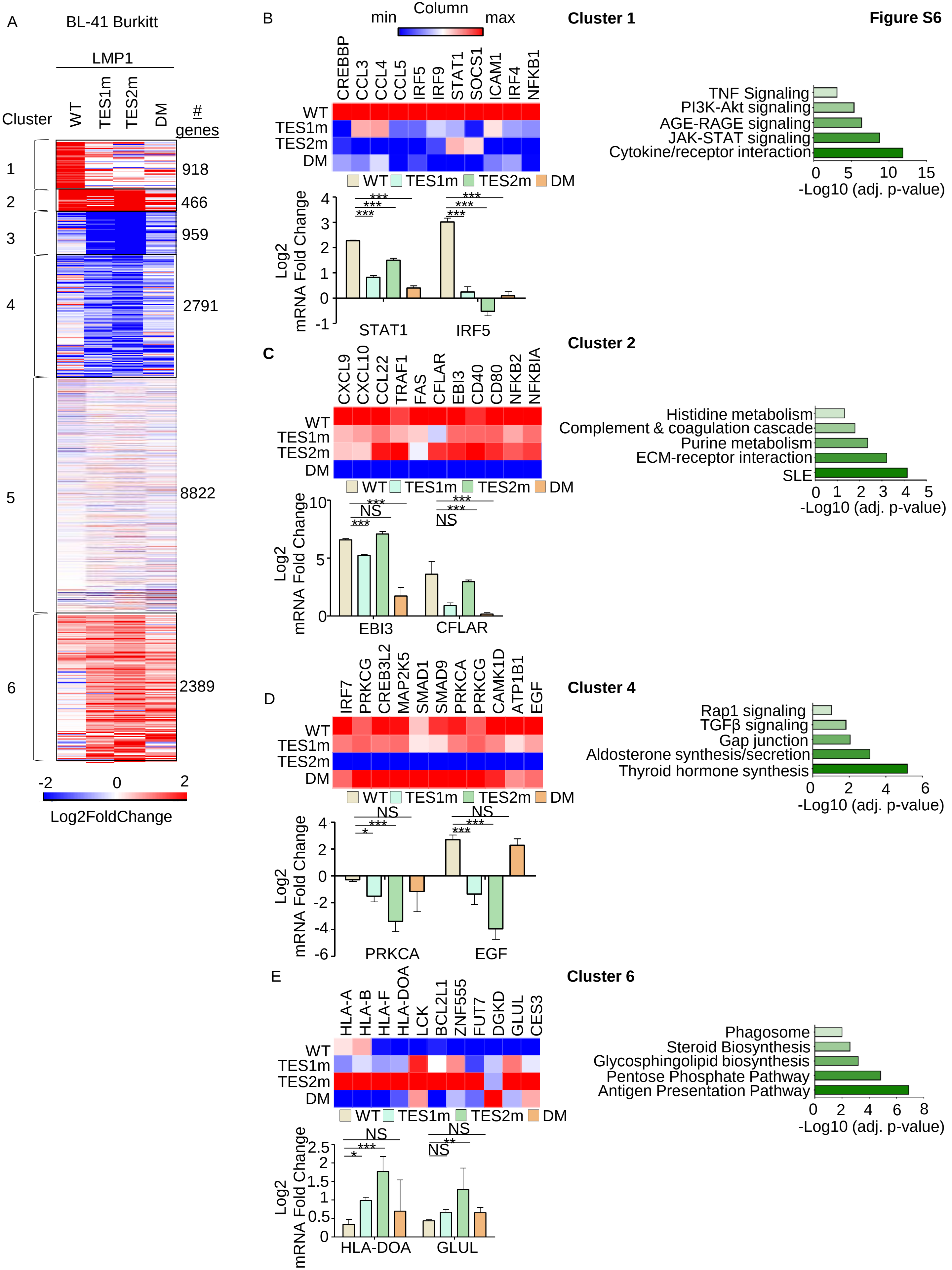
Figure S6
A
BL-41 Burkitt
LMP1
WT
TES1m
TES2m
DM
Cluster
genes
1
918
2
466
3
959
4
2791
5
8822
6
2389
-2
0
2
Log2FoldChange
B
Column
min
max
Cluster 1
CREBBP CCL3 CCL4 CCL5 IRF5 IRF9 STAT1 SOCS1 ICAM1 IRF4 NFKB1
WT TES1m TES2m DM
Log2 mRNA Fold Change
STAT1
IRF5
***
TNF Signaling
PI3K-Akt signaling
AGE-RAGE signaling
JAK-STAT signaling
Cytokine/receptor interaction
-Log10 (adj. p-value)
C
Cluster 2
CXCL9 CXCL10 CCL22 TRAF1 FAS CFLAR EBI3 CD40 CD80 NFKB2 NFKBIA
EBI3
CFLAR
NS
Histidine metabolism
Complement & coagulation cascade
Purine metabolism
ECM-receptor interaction
SLE
D
Cluster 4
IRF7 PRKCG CREB3L2 MAP2K5 SMAD1 SMAD9 PRKCA PRKCG CAMK1D ATP1B1 EGF
PRKCA
EGF
Rap1 signaling
TGFβ signaling
Gap junction
Aldosterone synthesis/secretion
Thyroid hormone synthesis
E
Cluster 6
HLA-A HLA-B HLA-F HLA-DOA LCK BCL2L1 ZNF555 FUT7 DGKD GLUL CES3
HLA-DOA
GLUL
Phagosome
Steroid Biosynthesis
Glycosphingolipid biosynthesis
Pentose Phosphate Pathway
Antigen Presentation Pathway

Fig. S6. RNAseq analysis of BL-41 B-cell responses to WT, TES1, TES2 or DM LMP1.

(A) K-means heatmap analysis of RNAseq datasets from n=3 replicates generated in EBV- BL-41 Burkitt cells with conditional LMP1 WT, TES1m, TES2m or DM expression induced by 250 ng/ml doxycycline for 24 hours. The heatmap visualizes host gene Log2 Fold change across the four conditions, divided into six clusters. A two-way ANOVA P value cutoff of <0.01 and >2-fold gene expression were used. # of genes in each cluster is indicated at right.

(B) Heatmaps of representative Cluster 1 differentially regulated genes (top), with column maximum (max) colored red and minimum (min) colored blue, as shown by the scalebar. Also shown are expression values of two representative Cluster 1 genes (lower left) and Enrichr analysis of KEGG pathways significantly enriched in Cluster 1 gene sets (lower right). p-values were determined by one-sided Fisher's exact test. ***p<0.001.

(C) Heatmaps of representative Cluster 2 differentially regulated genes (top). Also shown are expression values of two representative Cluster 2 genes (lower left) and Enrichr analysis of KEGG pathways significantly enriched in Cluster 2 gene sets (lower right). p-values were determined by one-sided Fisher's exact test. ***p<0.001.

(D) Heatmaps of representative Cluster 4 differentially regulated genes (top). Also shown are expression values of two representative Cluster 4 genes (lower left) and Enrichr analysis of KEGG pathways significantly enriched in Cluster 4 gene sets (lower right). p-values were determined by one-sided Fisher's exact test. *p<0.05, ***p<0.001.

(E) Heatmaps of representative Cluster 6 differentially regulated genes (top). Also shown are expression values of two representative Cluster 6 genes (lower left) and Enrichr analysis of KEGG pathways significantly enriched in Cluster 6 gene sets (lower right). p-values were determined by one-sided Fisher's exact test.*p<0.05, **p<0.01, ***p<0.001.

Figure S7

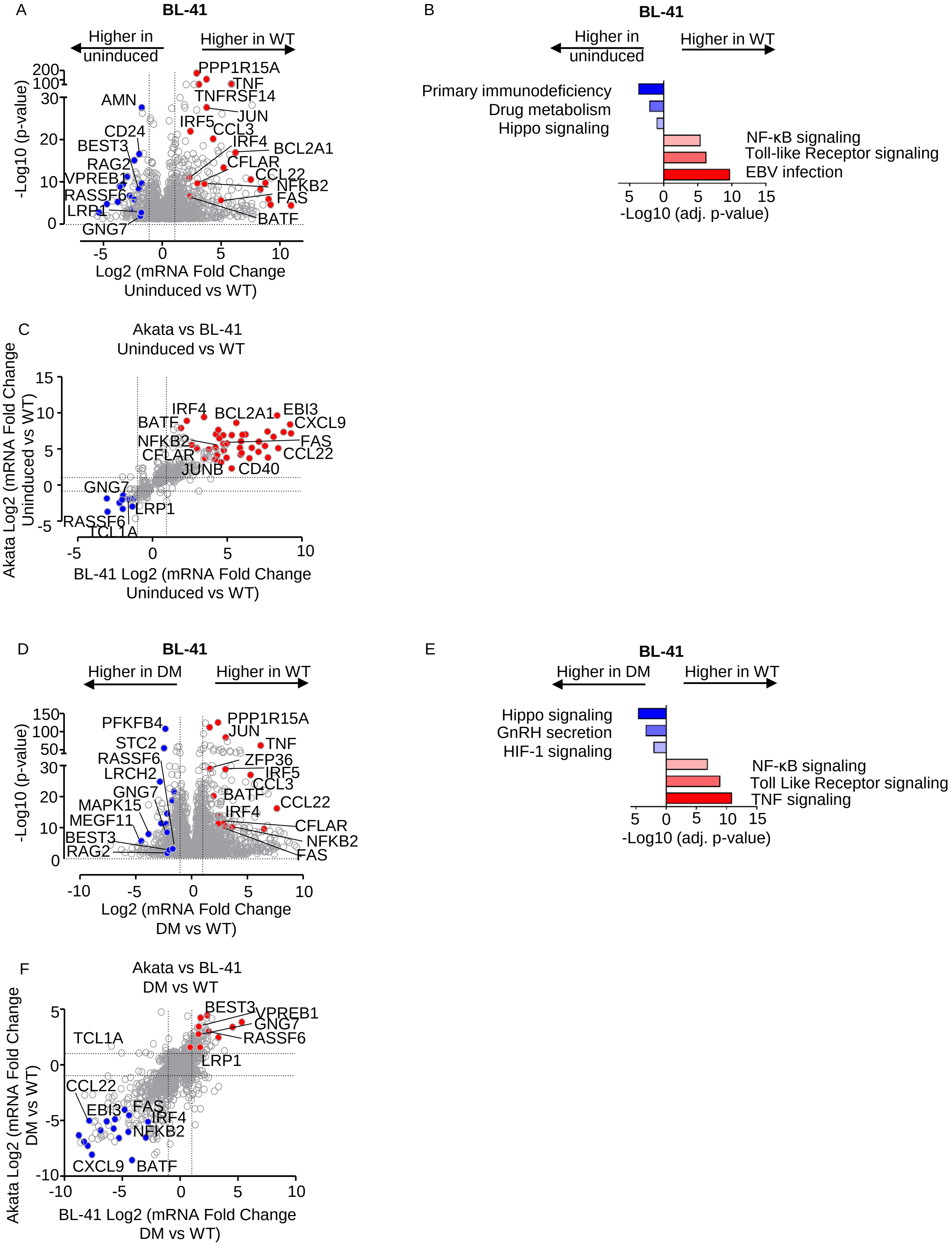
A
BL-41
Higher in uninduced
Higher in WT
PPP1R15A
TNF
TNFRSF14
JUN
AMN
IRF5
CCL3
CD24
IRF4
BCL2A1
BEST3
CFLAR
RAG2
CCL22
VPREB1
NFKB2
RASSF6
FAS
LRP1
BATF
GNG7
-Log10 (p-value)
Log2 (mRNA Fold Change Uninduced vs WT)
B
BL-41
Higher in uninduced
Higher in WT
Primary immunodeficiency
Drug metabolism
Hippo signaling
NF-κB signaling
Toll-like Receptor signaling
EBV infection
-Log10 (adj. p-value)
C
Akata vs BL-41
Uninduced vs WT
IRF4
BCL2A1
EBI3
BATF
CXCL9
NFKB2
FAS
CFLAR
CCL22
JUNB
CD40
GNG7
LRP1
RASSF6
TCL1A
Akata Log2 (mRNA Fold Change Uninduced vs WT)
BL-41 Log2 (mRNA Fold Change Uninduced vs WT)
D
BL-41
Higher in DM
Higher in WT
PFKFB4
PPP1R15A
JUN
STC2
TNF
RASSF6
ZFP36
LRCH2
IRF5
CCL3
GNG7
BATF
MAPK15
CCL22
IRF4
MEGF11
CFLAR
BEST3
NFKB2
RAG2
FAS
-Log10 (p-value)
Log2 (mRNA Fold Change DM vs WT)
E
BL-41
Higher in DM
Higher in WT
Hippo signaling
GnRH secretion
HIF-1 signaling
NF-κB signaling
Toll Like Receptor signaling
TNF signaling
-Log10 (adj. p-value)
F
Akata vs BL-41
DM vs WT
BEST3
VPREB1
GNG7
TCL1A
RASSF6
LRP1
CCL22
EBI3
FAS
IRF4
NFKB2
CXCL9
BATF
Akata Log2 (mRNA Fold Change DM vs WT)
BL-41 Log2 (mRNA Fold Change DM vs WT)

Fig. S7. Cross-comparison of WT and DM LMP1 effects on Akata vs BL-41 transcriptomes.

(A) Volcano plot analysis of host transcriptome-wide genes differentially expressed in BL-41 cells conditionally induced for WT LMP1 expression for 24h by 250 ng/ml Dox versus in mock induced cells. Higher X-axis fold changes indicate genes more highly expressed in cells with WT LMP1 expression, whereas lower X-axis fold changes indicate higher expression in cells mock induced for LMP1. Data are from n=3 RNAseq datasets.

(B) Enrichr analysis of KEGG pathways most highly enriched in RNAseq data as in (A) amongst genes more highly expressed in BL-41 with WT LMP1 (red) vs amongst genes more highly expressed with mock LMP1 induction (blue).

(C) Volcano plot cross-comparison of Log2 transformed fold change of host mRNA levels in BL-41 cells (X-axis) versus Akata cells (Y-axis) uninduced versus induced for WT LMP1 by 250 ng/ml Dox for 24 hours. Selected genes highly WT LMP1 induced in both Burkitt contexts are highlighted in red, whereas selected genes suppressed by LMP1 in both Burkitt contexts are highlighted in blue.

(D) Volcano plot analysis of host transcriptome-wide genes differentially expressed in BL-41 cells conditionally induced for DM versus WT LMP1 expression for 24h by 250 ng/ml Dox. Higher X-axis fold changes indicate genes more highly expressed in cells with WT LMP1 expression, whereas lower X-axis fold changes indicate higher expression in cells induced for DM LMP1. Data are from n=3 RNAseq datasets.

(B) Enrichr analysis of KEGG pathways most highly enriched in RNAseq data as in (D) amongst genes more highly expressed in BL-41 with WT LMP1 (red) vs amongst genes more highly expressed with DM LMP1 induction (blue).

(C) Volcano plot cross-comparison of Log2 transformed fold change of host mRNA levels in BL-41 cells (X-axis) versus Akata cells (Y-axis) induced for DM versus WT LMP1 by 250 ng/ml Dox for 24 hours. Selected genes highly WT LMP1 induced in both Burkitt contexts relative to levels in cells with DM LMP1 expression are highlighted in red, whereas selected genes suppressed by WT LMP1 in both Burkitt contexts are highlighted in blue.

Figure S8

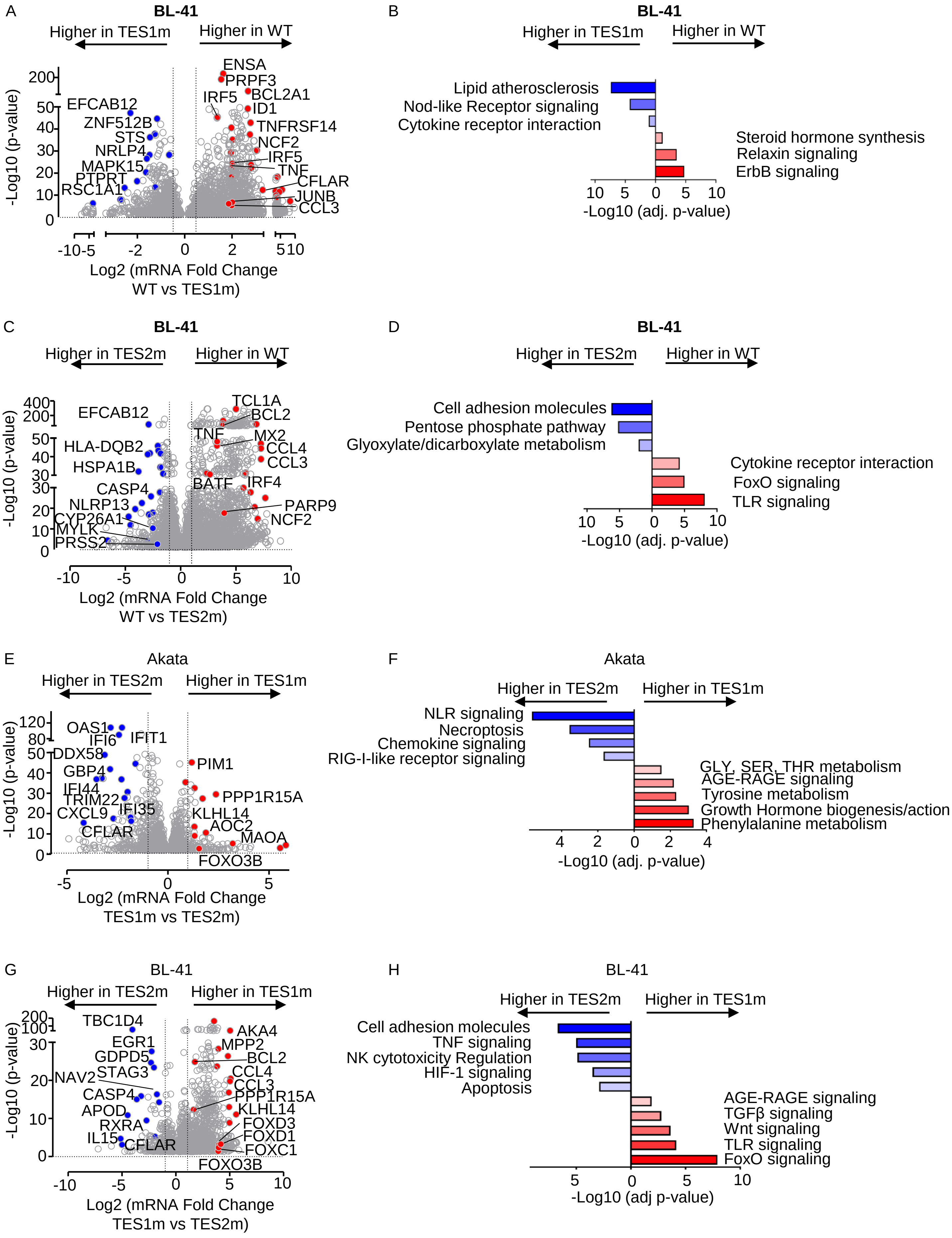
A
BL-41
Higher in TES1m
Higher in WT
ENSA
PRPF3
BCL2A1
IRF5
ID1
EFCAB12
ZNF512B
TNFRSF14
STS
NCF2
NRLP4
IRF5
MAPK15
TNF
PTPRT
CFLAR
RSC1A1
JUNB
CCL3
-Log10 (p-value)
200
50
40
30
20
10
0
-10-5
-2
0
2
510
Log2 (mRNA Fold Change
WT vs TES1m)
B
BL-41
Higher in TES1m
Higher in WT
Lipid atherosclerosis
Nod-like Receptor signaling
Cytokine receptor interaction
Steroid hormone synthesis
Relaxin signaling
ErbB signaling
10 5 0 5 10
-Log10 (adj. p-value)
C
BL-41
Higher in TES2m
Higher in WT
TCL1A
BCL2
EFCAB12
TNF
MX2
HLA-DQB2
CCL4
CCL3
HSPA1B
BATF
IRF4
CASP4
NLRP13
PARP9
CYP26A1
NCF2
MYLK
PRSS2
-Log10 (p-value)
400
200
50
40
30
30
20
10
0
-10
-5
0
5
10
Log2 (mRNA Fold Change
WT vs TES2m)
D
BL-41
Higher in TES2m
Higher in WT
Cell adhesion molecules
Pentose phosphate pathway
Glyoxylate/dicarboxylate metabolism
Cytokine receptor interaction
FoxO signaling
TLR signaling
10 5 0 5 10
-Log10 (adj. p-value)
E
Akata
Higher in TES2m
Higher in TES1m
OAS1
IFI6
IFIT1
DDX58
PIM1
GBP4
IFI44
TRIM22
PPP1R15A
IFI35
CXCL9
KLHL14
AOC2
CFLAR
MAOA
FOXO3B
-Log10 (p-value)
120
80
50
40
30
20
10
0
-5
0
5
Log2 (mRNA Fold Change
TES1m vs TES2m)
F
Akata
Higher in TES2m
Higher in TES1m
NLR signaling
Necroptosis
Chemokine signaling
RIG-I-like receptor signaling
GLY, SER, THR metabolism
AGE-RAGE signaling
Tyrosine metabolism
Growth Hormone biogenesis/action
Phenylalanine metabolism
4 2 0 2 4
-Log10 (adj. p-value)
G
BL-41
Higher in TES2m
Higher in TES1m
TBC1D4
AKA4
EGR1
MPP2
GDPD5
BCL2
STAG3
CCL4
NAV2
CCL3
CASP4
PPP1R15A
APOD
KLHL14
RXRA
FOXD3
IL15
FOXD1
CFLAR
FOXC1
FOXO3B
-Log10 (p-value)
200
100
30
20
10
0
-10
-5
0
5
10
Log2 (mRNA Fold Change
TES1m vs TES2m)
H
BL-41
Higher in TES2m
Higher in TES1m
Cell adhesion molecules
TNF signaling
NK cytotoxicity Regulation
HIF-1 signaling
Apoptosis
AGE-RAGE signaling
TGFβ signaling
Wnt signaling
TLR signaling
FoxO signaling
5 0 5 10
-Log10 (adj p-value)

Fig. S8. Cross-comparison of TES1 and TES2 LMP1 effects on Akata vs BL-41 transcriptomes.

A) Volcano plot analysis of host transcriptome-wide genes differentially expressed in BL-41 cells conditionally induced for TES1m vs WT LMP1 expression for 24h by 250 ng/ml Dox. Higher X-axis fold changes indicate genes more highly expressed in cells with WT LMP1 expression, whereas lower X-axis fold changes indicate higher expression induced for TES1m LMP1. Data are from n=3 RNAseq datasets.

(B) Enrichr analysis of KEGG pathways most highly enriched in RNAseq data as in (A) amongst genes more highly expressed in BL-41 with WT LMP1 (red) vs amongst genes more highly expressed with TES1m LMP1 induction (blue).

(C) Volcano plot analysis of host transcriptome-wide genes differentially expressed in BL-41 cells conditionally induced for TES2m vs WT LMP1 expression for 24h by 250 ng/ml Dox. Higher X-axis fold changes indicate genes more highly expressed in cells with WT LMP1 expression, whereas lower X-axis fold changes indicate higher expression induced for TES2m LMP1. Data are from n=3 RNAseq datasets.

(D) Enrichr analysis of KEGG pathways most highly enriched in RNAseq data as in (A) amongst genes more highly expressed in BL-41 with WT LMP1 (red) vs amongst genes more highly expressed with TES2m LMP1 induction (blue).

(E) Volcano plot analysis of host transcriptome-wide genes differentially expressed in Akata cells conditionally induced for TES1m vs TES2m LMP1 expression for 24h by 250 ng/ml Dox. Higher X-axis fold changes indicate genes more highly expressed in cells with TES1m LMP1 expression, whereas lower X-axis fold changes indicate higher expression induced for TES2m LMP1. Data are from n=3 RNAseq datasets.

(F) Enrichr analysis of KEGG pathways most highly enriched in RNAseq data as in (E) amongst genes more highly expressed in Akata with TES1m LMP1 (red) vs amongst genes more highly expressed with TES2m LMP1 induction (blue).

(G) Volcano plot analysis of host transcriptome-wide genes differentially expressed in BL-41 cells conditionally induced for TES1m vs TES2m LMP1 expression for 24h by 250 ng/ml Dox. Higher X-axis fold changes indicate genes more highly expressed in cells with TES1m LMP1 expression, whereas lower X-axis fold changes indicate higher expression induced for TES2m LMP1. Data are from n=3 RNAseq datasets.

(B) Enrichr analysis of KEGG pathways most highly enriched in RNAseq data as in (G) amongst genes more highly expressed in BL-41 with TES1m LMP1 (red) vs amongst genes more highly expressed with TES2m LMP1 induction (blue).

Figure S9

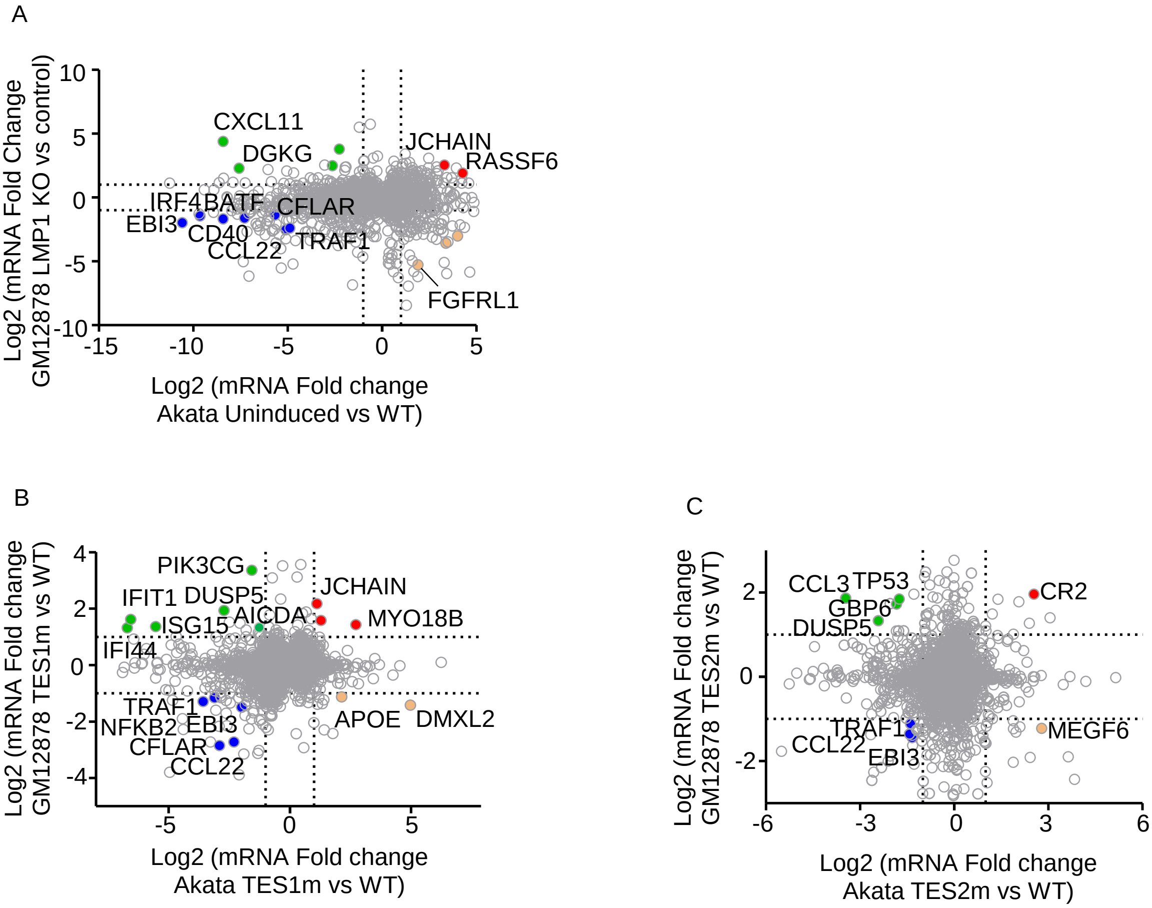
A
Log2 (mRNA Fold Change
GM12878 LMP1 KO vs control)
Log2 (mRNA Fold change
Akata Uninduced vs WT)
10
5
0
-5
-10
-15
-10
-5
0
5
CXCL11
DGKG
JCHAIN
RASSF6
IRF4
BATF
CFLAR
EBI3
CD40
CCL22
TRAF1
FGFRL1
B
Log2 (mRNA Fold change
GM12878 TES1m vs WT)
Log2 (mRNA Fold change
Akata TES1m vs WT)
4
2
0
-2
-4
-5
0
5
PIK3CG
JCHAIN
IFIT1
DUSP5
AICDA
MYO18B
ISG15
IFI44
TRAF1
NFKB2
EBI3
APOE
DMXL2
CFLAR
CCL22
C
Log2 (mRNA Fold change
GM12878 TES2m vs WT)
Log2 (mRNA Fold change
Akata TES2m vs WT)
2
0
-2
-6
-3
0
3
6
CCL3
TP53
CR2
GBP6
DUSP5
TRAF1
CCL22
EBI3
MEGF6

Figure S9. Cross-comparison of host genes differentially expressed upon perturbation of LCL LMP1 versus upon LMP1 induction in Akata cells.

(A) Volcano plot analysis of host genes differentially expressed upon WT LMP1 induction in Akata (X-axis) versus upon LMP1 KO in GM12878 (Y-axis). Shown are Log2 transformed mRNA fold change values for Akata cells mock induced versus induced for LMP1 WT expression for 24 hours (X-axis) versus upon expression of LMP1 vs control sgRNA in GM18278 for 48 hours. Genes more highly expressed in mock-induced Akata have higher x-axis values, whereas genes more highly expressed in Akata induced for WT LMP1 have lower x-axis values. Likewise, genes with higher expression with control sgRNA expression have higher y-axis values, whereas genes with lower expression in GM12878 with LMP1 KO have lower Y-axis values. P value <0.05 and >2-fold gene expression cutoffs were used.

(B) Volcano plot analysis of host genes differentially expressed upon TES1m vs WT LMP1 induction in Akata (X-axis) versus upon rescue of LMP1 KO GM12878 with TES1m versus WT LMP1 (Y-axis). Shown are Log2 transformed mRNA fold change values for Akata cells induced for TES1m versus WT LMP1 expression for 24 hours (X-axis) versus upon rescue of GM18278 LMP1 KO with TES1m vs WT LMP1 cDNA, as in **Fig 3**. Genes more highly expressed in Akata with TES1m than WT LMP1 expression have higher x-axis values, whereas genes more highly expressed in Akata induced for WT LMP1 than TES1m have lower x-axis values. Likewise, GM12878 genes with higher expression with TES1m rescue have higher y-axis values, whereas genes with lower expression in GM12878 with TES1m than WT rescue have lower Y-axis values. P value <0.05 and >2-fold gene expression cutoffs were used.

(C) Volcano plot analysis of host genes differentially expressed upon TES2m vs WT LMP1 induction in Akata (X-axis) versus upon rescue of LMP1 KO GM12878 with TES2m versus WT LMP1 (Y-axis). Shown are Log2 transformed mRNA fold change values for Akata cells induced for TES2m versus WT LMP1 expression for 24 hours (X-axis) versus upon rescue of GM18278 LMP1 KO with TES2m vs WT LMP1 cDNA, as in **Fig 3**. Genes more highly expressed in Akata with TES2m than WT LMP1 expression have higher x-axis values, whereas genes more highly expressed in Akata induced for WT LMP1 than TES2m have lower x-axis values. Likewise, GM12878 genes with higher expression with TES2m rescue have higher y-axis values, whereas genes with lower expression in GM12878 with TES2m than WT rescue have lower Y-axis values. P value <0.05 and >2-fold gene expression cutoffs were used.

Figure S10

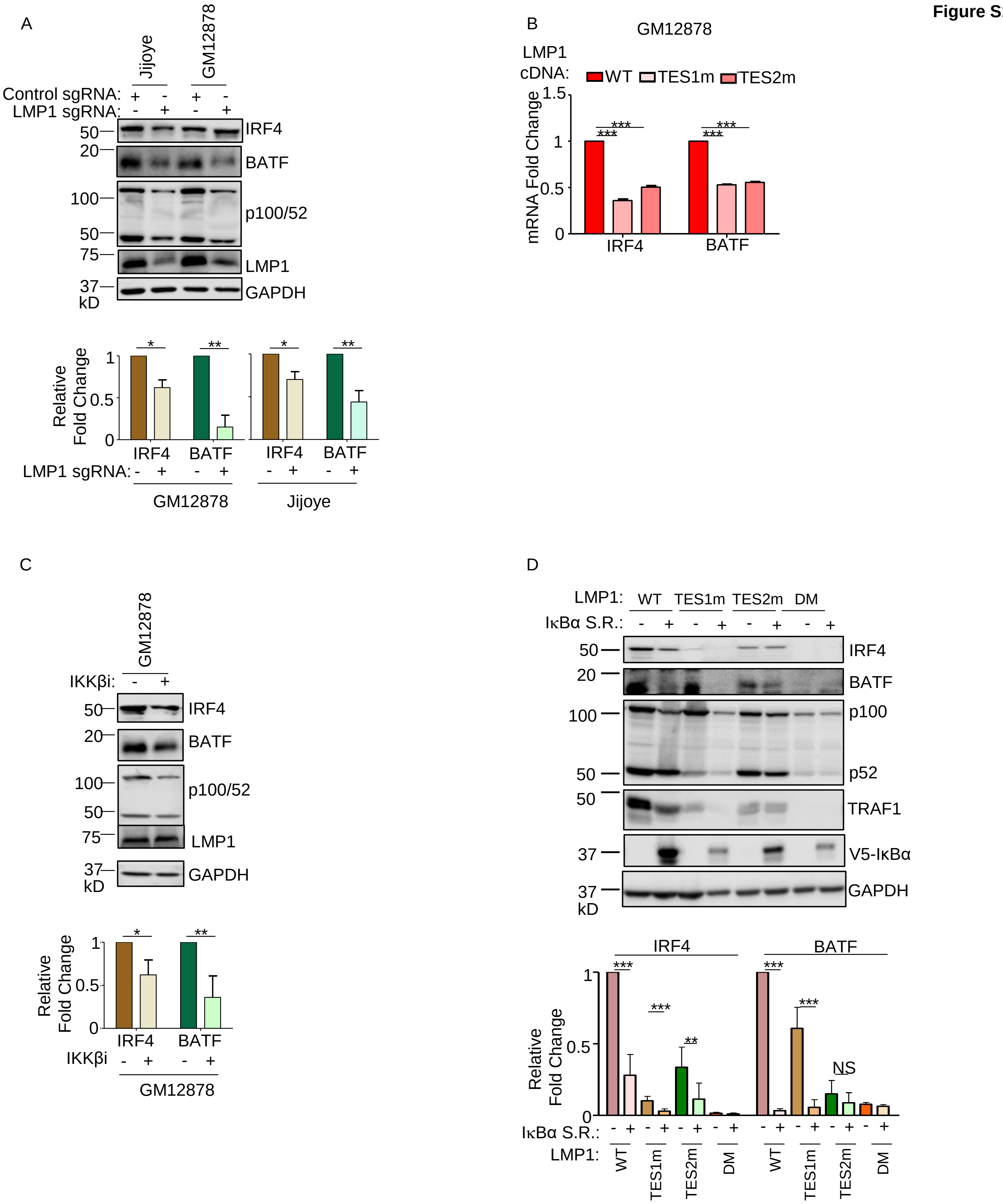
A
Jijoye
GM12878
Control sgRNA: + - + -
LMP1 sgRNA: - + - +
IRF4
BATF
p100/52
LMP1
GAPDH
kD
Relative Fold Change
IRF4
BATF
LMP1 sgRNA:
GM12878
Jijoye
B
GM12878
LMP1 cDNA:
WT
TES1m
TES2m
mRNA Fold Change
IRF4
BATF
C
GM12878
IKKβi:
IRF4
BATF
p100/52
LMP1
GAPDH
kD
Relative Fold Change
IKKβi
D
LMP1:
WT
TES1m
TES2m
DM
IκBα S.R.:
IRF4
BATF
p100
p52
TRAF1
V5-IκBα
GAPDH
kD
Relative Fold Change
NS

Fig. S10. Roles of TES1 and TES2 canonical NF-κB pathways in BATF and IRF4 induction.

(A) Immunoblot analysis of WCL from latency III Jijoye Burkitt cells or GM12878 LCL expressing LMP1 targeting sgRNA, as indicated. Shown below are relative fold changes + SD from n=3 replicates of IRF4 or BATF vs GAPDH load control densitometry values, with values in sgRNA control expressing cells set to 1. P-values were determined by one-sided Fisher's exact test. **$p<0.001$, ***$p<0.0001$.

(B) Mean + SD fold changes of IRF4 and BATF mRNA abundances from n=3 RNAseq replicates of GM12878 LCLs transduced with lentivirus expressing LMP1 sgRNA and induced for WT, TES1m or TES2m rescue cDNA expression for 6 days, as in Fig 3. P-values were determined by one-sided Fisher's exact test. ***$p<0.001$.

(C) Immunoblot analysis of WCL from GM12878 LCLs treated with vehicle control or 1 μM IKKβ inhibitor VIII for 24 hours. Shown below are relative foldchanges + SD from n=3 replicates of IRF4 or BATF vs GAPDH load control densitometry values. Levels in vehicle control treated WT LMP1 expressing cells were set to 1. P-values were determined by one-sided Fisher's exact test. **$p<0.001$, ***$p<0.0001$.

(D) Immunoblot analysis of WCL from Akata cells induced for the indicated LMP1 construct expression, either without or together with an IκBα super-repressor (IκBα-S.R.) that blocks canonical NF-κB signaling. Shown below are relative foldchanges + SD of IRF4 or BATF vs GAPDH load control densitometry values from n=3 replicates, with values in cells expressing WT LMP1 but not IκBα- set to 1. P-values were determined by one-sided Fisher's exact test. **$p<0.001$, ***$p<0.0001$.

Figure S11

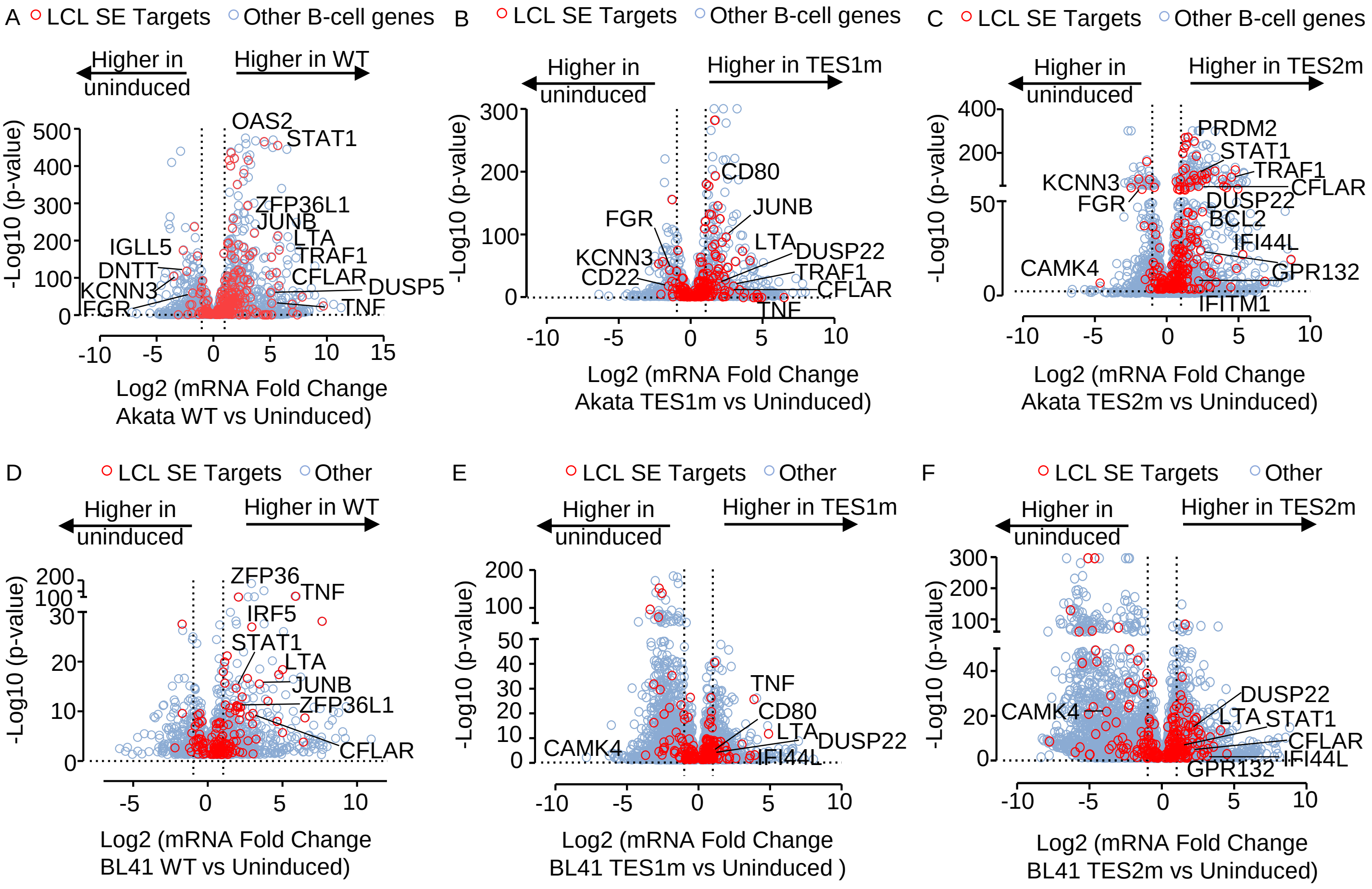

A
LCL SE Targets
Other B-cell genes
Higher in uninduced
Higher in WT
OAS2
STAT1
ZFP36L1
JUNB
LTA
TRAF1
CFLAR
DUSP5
TNF
IGLL5
DNTT
KCNN3
FGR
-Log10 (p-value)
Log2 (mRNA Fold Change Akata WT vs Uninduced)
B
LCL SE Targets
Other B-cell genes
Higher in uninduced
Higher in TES1m
CD80
JUNB
FGR
LTA
DUSP22
TRAF1
CFLAR
TNF
KCNN3
CD22
-Log10 (p-value)
Log2 (mRNA Fold Change Akata TES1m vs Uninduced)
C
LCL SE Targets
Other B-cell genes
Higher in uninduced
Higher in TES2m
PRDM2
STAT1
TRAF1
CFLAR
KCNN3
FGR
DUSP22
BCL2
IFI44L
GPR132
CAMK4
IFITM1
-Log10 (p-value)
Log2 (mRNA Fold Change Akata TES2m vs Uninduced)
D
LCL SE Targets
Other
Higher in uninduced
Higher in WT
ZFP36
TNF
IRF5
STAT1
LTA
JUNB
ZFP36L1
CFLAR
-Log10 (p-value)
Log2 (mRNA Fold Change BL41 WT vs Uninduced)
E
LCL SE Targets
Other
Higher in uninduced
Higher in TES1m
TNF
CD80
LTA
DUSP22
IFI44L
CAMK4
-Log10 (p-value)
Log2 (mRNA Fold Change BL41 TES1m vs Uninduced)
F
LCL SE Targets
Other
Higher in uninduced
Higher in TES2m
DUSP22
LTA
STAT1
CFLAR
IFI44L
GPR132
CAMK4
-Log10 (p-value)
Log2 (mRNA Fold Change BL41 TES2m vs Uninduced)

Figure S11. LMP1 TES1 and TES2 roles in EBV super-enhancer target gene regulation in Akata and BL-41 Burkitt B-cells.

(A) Volcano plot analysis of Akata RNA-seq, comparing mRNA values in cells induced for LMP1 WT vs. mock-induced for 24 hours. SE targeted genes highlighted by red circles and other B-cell genes indicated by blue circles. P value <0.05 and >2-fold gene expression cutoffs were used.

(B) Volcano plot analysis of Akata RNA-seq, comparing mRNA values in cells induced for LMP1 TES1m vs. mock-induced for 24 hours. SE targeted genes highlighted by red circles and other B-cell genes indicated by blue circles. P value <0.05 and >2-fold gene expression cutoffs were used.

(C) Volcano plot analysis of Akata RNA-seq, comparing mRNA values in cells induced for LMP1 TES2m vs. mock-induced for 24 hours. SE targeted genes highlighted by red circles and other B-cell genes indicated by blue circles. P value <0.05 and >2-fold gene expression cutoffs were used.

(D) Volcano plot analysis of BL-41 RNA-seq, comparing mRNA values in cells induced for LMP1 WT vs. mock-induced for 24 hours. SE targeted genes highlighted by red circles and other B-cell genes indicated by blue circles. P value <0.05 and >2-fold gene expression cutoffs were used.

(E) Volcano plot analysis of BL-41 RNA-seq, comparing mRNA values in cells induced for LMP1 TES1m vs. mock-induced for 24 hours. SE targeted genes highlighted by red circles and other B-cell genes indicated by blue circles. P value <0.05 and >2-fold gene expression cutoffs were used.

(F) Volcano plot analysis of BL-41 RNA-seq, comparing mRNA values in cells induced for LMP1 TES2m vs. mock-induced for 24 hours. SE targeted genes highlighted by red circles and other B-cell genes indicated by blue circles. P value <0.05 and >2-fold gene expression cutoffs were used.
